# Cortical field maps across human sensory cortex

**DOI:** 10.3389/fncom.2023.1232005

**Published:** 2023-12-15

**Authors:** Alyssa A. Brewer, Brian Barton

**Affiliations:** ^1^mindSPACE Laboratory, Departments of Cognitive Sciences and Language Science (by Courtesy), Center for Hearing Research, University of California, Irvine, Irvine, CA, United States; ^2^mindSPACE Laboratory, Department of Cognitive Sciences, University of California, Irvine, Irvine, CA, United States

**Keywords:** visual field map, auditory field map, cloverleaf cluster, retinotopy, tonotopy, periodotopy, somatotopy, gustatory

## Abstract

Cortical processing pathways for sensory information in the mammalian brain tend to be organized into topographical representations that encode various fundamental sensory dimensions. Numerous laboratories have now shown how these representations are organized into numerous cortical field maps (CMFs) across visual and auditory cortex, with each CFM supporting a specialized computation or set of computations that underlie the associated perceptual behaviors. An individual CFM is defined by two orthogonal topographical gradients that reflect two essential aspects of feature space for that sense. Multiple adjacent CFMs are then organized across visual and auditory cortex into macrostructural patterns termed cloverleaf clusters. CFMs within cloverleaf clusters are thought to share properties such as receptive field distribution, cortical magnification, and processing specialization. Recent measurements point to the likely existence of CFMs in the other senses, as well, with topographical representations of at least one sensory dimension demonstrated in somatosensory, gustatory, and possibly olfactory cortical pathways. Here we discuss the evidence for CFM and cloverleaf cluster organization across human sensory cortex as well as approaches used to identify such organizational patterns. Knowledge of how these topographical representations are organized across cortex provides us with insight into how our conscious perceptions are created from our basic sensory inputs. In addition, studying how these representations change during development, trauma, and disease serves as an important tool for developing improvements in clinical therapies and rehabilitation for sensory deficits.

## 1 Introduction

Topographical representations of sensory information are emerging as a fundamental organizational pattern for perceptual processing across sensory cortex in numerous mammalian species (Kaas, 1997; Wandell et al., 2005; Krubitzer, 2007; Sanchez-Panchuelo et al., 2010; Barton et al., 2012; Prinster et al., 2017; Yushu Chen et al., 2021). Organized topographies within sensory pathways are thought to support the comparison and combination of the information carried by the various specialized neuronal populations. To enhance the brain’s ability to discriminate among different stimuli, sensory neurons that respond to similar features are frequently organized into distinct clusters or columns, and their response characteristics exhibit smooth transitions across the cortical surface. The orderly connectivity arising from such organization is likely important for increasing the efficiency of such local processes as lateral inhibition and gain control and may provide a framework for sensory processing across the sensory hierarchy (Mitchison, 1991; Van Essen, 2003; Chklovskii and Koulakov, 2004; Shapley et al., 2007; Moradi and Heeger, 2009).

**Figure 1:**
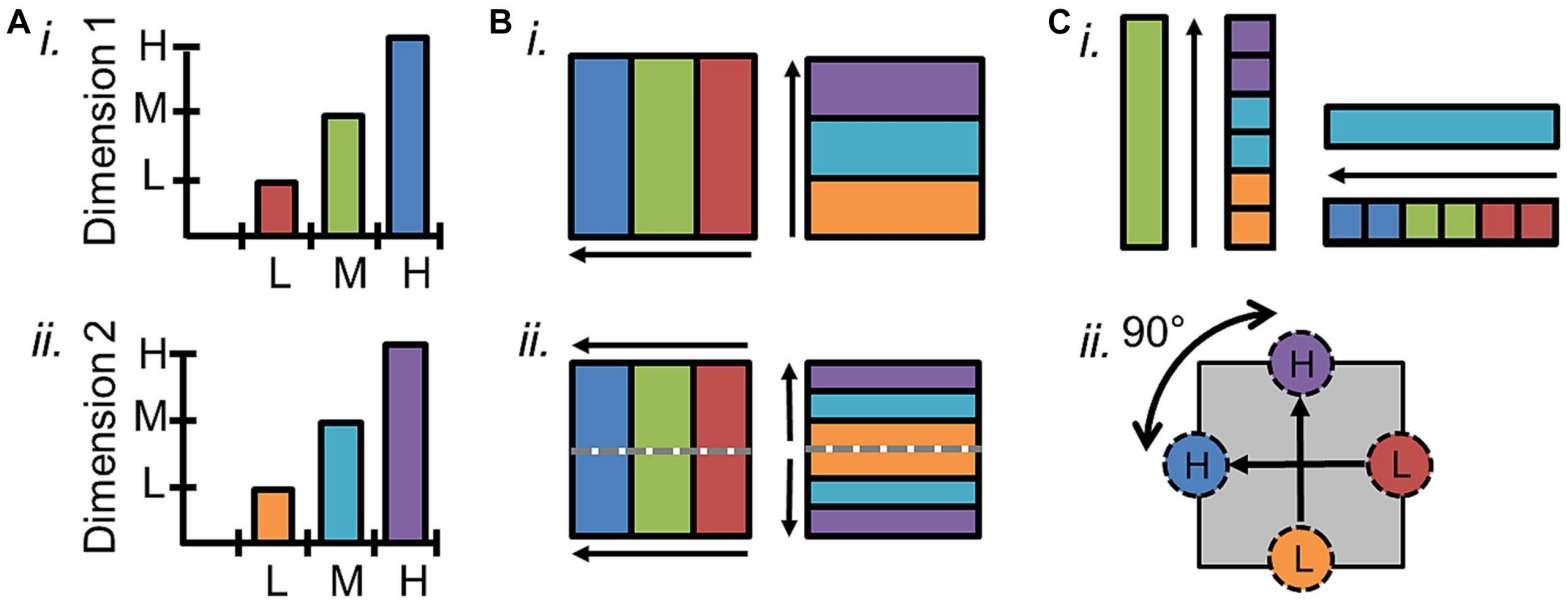
Definition of cortical field maps. **(A)** Schematics depict the two orthogonal dimensions that are required to define a cortical field map. **(i)** The graph of one sensory dimension (e.g., eccentricity; tonotopy) demonstrates measurements of three stimulus values—1: low (L, red); 2: medium (M, green); 3: high (H, blue). **(ii)** The graph of a second sensory dimension (e.g., polar angle; periodotopy) demonstrates measurements of three stimulus values—1: low (L, orange); 2: medium (M, cyan); 3: high (H, purple). **(B) (i)** Schematic depicts a single set of orthogonal gradients composing one CFM—one for each dimension in **(A)**. **(ii)** Schematic here demonstrates how a reversal in the dimension-2 gradient representations (*right*) divides up the single representation of the dimension-1 gradient (*left*) into two CFMs. Gray dotted lines show the boundary defined by the dimension-2 gradient reversal, and arrows denote the low-to-high gradients. **(C) (i)** In order for each voxel/portion of the CFM to represent a unique combination of dimension 1 and dimension 2 values, the two gradients composing a CFM must be orthogonal. In this case, measurements along the cortical representation of a single value (e.g., green, “M”) of dimension 1 span all values of dimension 2 (*right*), and vice versa (*left*). **(ii)** Diagram demonstrates how vectors drawn from centers of low-stimulus-value regions of interest (ROIs) to high-stimulus-value ROIs for each dimension should have an offset of approximately 90° in a CFM.

In human, the historically most-studied sensory topography is the representation of visual space in the visual system (Van Essen, 2003). Visual cortex contains multiple regions in which neurons are organized with respect to the neural arrangement of the retina, where neighboring photoreceptors respond to neighboring regions of visual space (Wandell et al., 2007). This organization serves as a map of visual space, also known as a visual field map (VFM), which repeats as an organizational pattern from the retina into higher-order visual processing (Engel et al., 1994; Sereno et al., 1995; DeYoe et al., 1996; Engel et al., 1997). Representing the fundamental visual dimensions of eccentricity (i.e., center-to-periphery) and polar angle (i.e., around-the-clock), a VFM is one form of a sensory cortical field map (CFM), a region which encodes at least two primary sensory dimensions (Figure 1; Engel et al., 1997; Wandell et al., 2007). More recent studies have revealed complete CFMs in human auditory cortex, with auditory field maps (AFMs) tiling human primary auditory core and belt regions, and partial CFM topographies in somatosensation (touch/pain) and gustation (taste), suggesting that CFMs serve as the building blocks of sensory processing (Murthy, 2011; Barton et al., 2012; Ma et al., 2012; Mancini et al., 2012; Brewer and Barton, 2016b; Prinster et al., 2017; Sanchez Panchuelo et al., 2018; Saadon-Grosman et al., 2020b; Willoughby et al., 2020). Understanding the characteristics of these CFMs, together with knowledge of the stimulus selectivity of the neurons within them, provides the foundation for understanding the specific computations carried out in particular sensory systems.

## 2 Cortical field map overview

### 2.1 CFM characteristics

**Figure 2:**
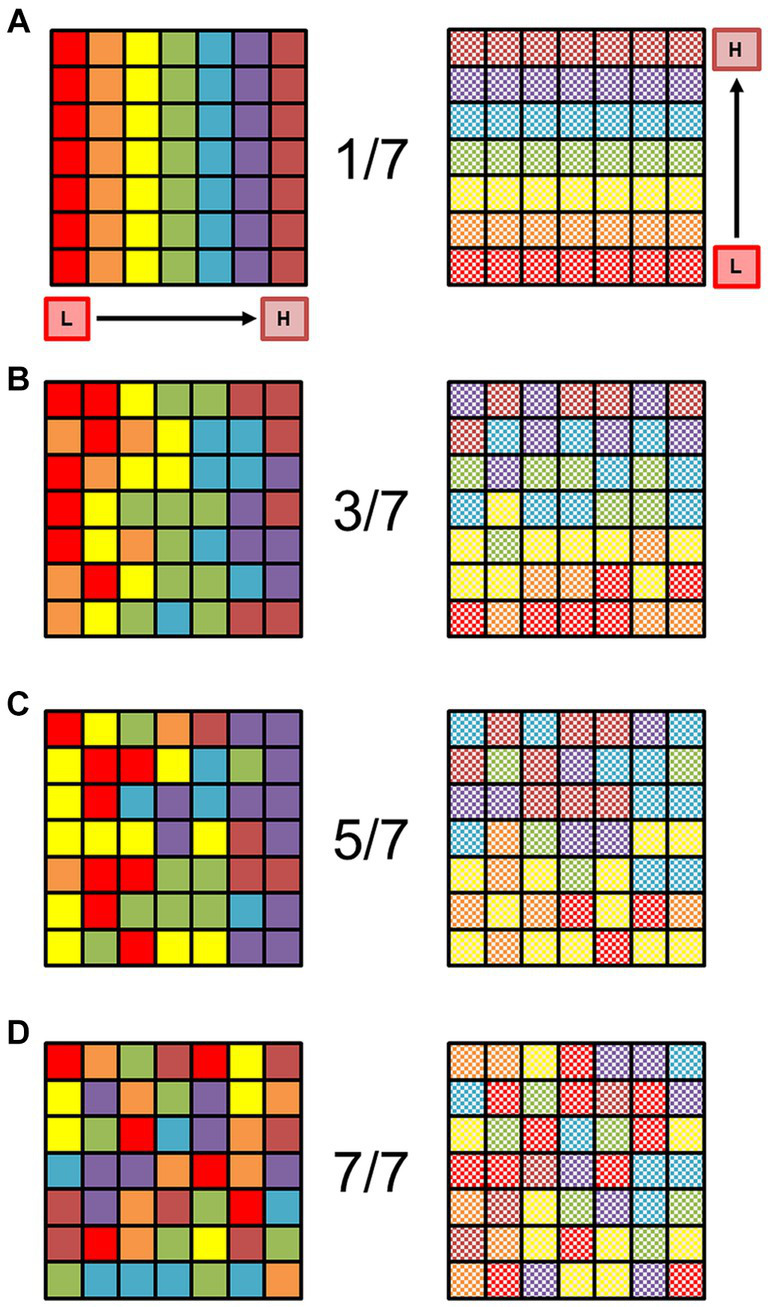
Organized orthogonal gradients of sensory representations are unlikely to occur by chance. **(A)** The square schematic represents a 7 × 7 matrix of voxels, in which each row represents one sensory gradient (red on the left through the rainbow to magenta on the right) evenly distributed across a piece of cortical surface *(left)*. Each color is supposed to represent a stimulus value spanning 1/7 of the full stimulus space for one dimension (e.g., eccentricity in vision or tonotopy in audition), with the lowest value of stimulus dimension 1 coded as red, and the highest value coded as magenta. For an example in the visual domain, the red squares would then represent voxels with a preference for eccentricity from fixation of 0.00°–1.57° of visual angle for an 11°—radius visual stimulus, green would be 4.71°–6.28°, and brown would be 9.43°–11.00°. No random noise has been added to this matrix; the colored squares represent a perfectly organized topographic map in cortex (Note that this is a level of perfection that does not exist in biological systems). Schematic square now represents the second, orthogonal dimension of the same sensory space (e.g., polar angle in vision or periodicity in audition; *right*). Organized and orthogonal gradients must be present for at least 2 sensory dimensions like this for the definition of a cortical field map. Note the regular gradient is still present with no noise, running from the low value of dimension 2 in checkered red (bottom of square) to checkered magenta (top of square). **(B)** Schematics of the same sensory gradients are now depicted more naturally with some noise added in. The random noise has been set so that if a voxel should represent a particular 1/7th of the stimulus range in the gradient [as seen in **(A)**], it can with equal probability represent an adjacent color. In other words, the “true” value falls somewhere within 3/7th of the stimulus range, centered on the correct value: if a voxel should be yellow in a perfect representation, the noise level would allow it to be orange, yellow, or green with equal probability. The overall direction of each orthogonal gradient is still mostly visible despite the noise. **(C)** Now the colors have been randomized so that each voxel can with equal probability represent 5/7th of the stimulus range, centered on the correct value (e.g., if a voxel should be yellow, it could be red, orange, yellow, green, or blue, with equal probability). The low-to-high directions of the two gradients are not very apparent, but there is a loose grouping of lower and higher stimulus values on each side. **(D)** Each voxel has now been randomly assigned to any of the 7 colors with equal probability. No stimulus gradient structure is present. Adapted from Barton and Brewer (2017), licensed under CC BY.

Accurate identification of individual CFMs is essential for parsing the individual computational stages of sensory processing. Several characteristics are necessary to establish that a particular cortical representation is a CFM and define its borders. First, the topographical representations of each sensory dimension should be organized as an orderly gradient covering a contiguous range of that dimension (Figures 1A,B; Brewer and Barton, 2018). Such a topographical gradient typically represents one aspect of either a peripheral sensing organ (i.e., visual eccentricity across the retina or auditory tonotopy along the basilar membrane of the inner ear) or another important dimension of sensory features (i.e., periodicity in audition). While care must be taken to correctly identify these gradients in fMRI measurements, such organized responses are exceedingly unlikely to emerge in fMRI measurements by chance (Figure 2; for further discussion, see Barton and Brewer, 2017).

The representation of an individual sensory dimension often can appear as a wide swath of topographical responses across a region of sensory cortex. For example, the representation of visual eccentricity along the occipital pole spreads across the region as a contiguous, apparently unified gradient. Without other markers, it is impossible to determine how such a representation would be divided into the individual CFMs that tile this region and contribute to specific sensory processing steps. Thus, it is important to distinguish between a single topographical gradient and a complete CFM. Simply guessing at how to divide up a single topographical gradient based on factors like anatomical location, data averaged across subjects, or diagrams from homologous monkey data to complete the perpendicular boundaries, as has frequently been done for tonotopic measurements in human auditory cortex, is not at all sufficient in most cases (for detailed review, see Brewer and Barton, 2016b; for example exceptions, see Formisano et al., 2003; Hinds et al., 2008; Benson et al., 2012). A CFM must be defined by the presence of two overlapping topographical gradients that each represent a different, orthogonal sensory dimension – i.e., visual eccentricity and polar angle or auditory tonotopy and periodotopy (Figure 1; Brewer et al., 2005; Wandell et al., 2007; Barton et al., 2012; Brewer and Barton, 2012). Figure 1A demonstrates a schematic of two orthogonal representations that form a single CFM (Figure 1Bi), while Figure 1Bii shows how a matching representation of dimension 2 can be divided into two CFMs based on the reversal of dimension 1 at the dotted gray line. The measurement of a single gradient across a region of cortex could thus denote a single CFM or many CFMs. As the number of overlapping orthogonal gradients increases, the determination of the CFM organization grows increasingly complex. Cortical regions may even be composed of topographical gradients representing several sensory dimensions, such as the representations of spatial frequency and orientation selectivity that are present in primary visual cortex along with the retinotopic representations of visual space. Thus any two sets of representations of orthogonal sensory dimensions at minimum can be used to define a CFM.

In addition to its two-dimensional (2D) orthogonality (Figure 1C), the internal topography of each CFM should be non-repeating; the computation for a particular CFM should be performed across a single region of the sensory domain (Sereno et al., 1995; DeYoe et al., 1996; Press et al., 2001; Wandell et al., 2007; Barton et al., 2012). Similarly, each CFM should represent a considerable portion of the sensory dimensions, although increases in the magnification of specific parts of sensory space, like seen for the central fovea in the visual system, and limitations in measurement resolution may both reduce the measurable range. Finally, while some variation is expected across individuals, the basic overall layout and composition of CFMs should be reliably consistent. CFMs even in low-order visual and auditory cortex can differ substantially in size and anatomical location, but the overall arrangement of adjacent CFMs should be maintained across individuals (Galaburda and Sanides, 1980; Rademacher et al., 1993; Morosan et al., 2001; Rademacher et al., 2001; Schonwiesner et al., 2002; Dougherty et al., 2003; Brewer et al., 2005; Wandell et al., 2007; Clarke and Morosan, 2012).

### 2.2 CFM boundaries

**Figure 3:**
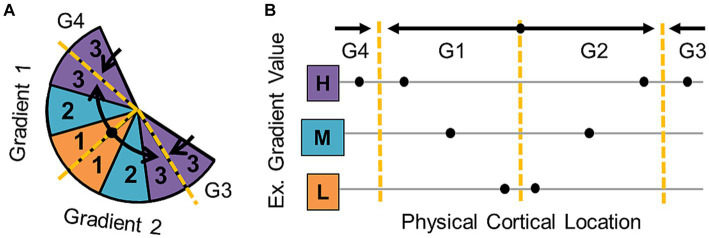
Cortical field map boundary definitions. **(A)** Diagram represents the organization of a series of gradients of one sensory dimension (e.g., polar angle—vision; periodotopy—auditory) along a flattened cortical surface. Black arrows denote the gradient directions—low (orange) to medium (cyan) to high (purple). Dashed yellow lines mark gradient “reversals” that are used to define the boundaries between individual cortical field maps. **(B)** The schematic illustrates how gradient boundaries for one dimension of a CFM are defined at sections where the gradient reverses direction. Hypothetical measurement points along the cortical surface of a region of interest (ROI) are shown as black dots. Black arrows demonstrate the low-to-high direction of each gradient, and dotted yellow lines mark the reversals that separate the data points into four separate gradients (G1, G2, G3, G4).

Using these characteristics to accurately define the boundaries of specific CFMs is key for isolating individual stages of sensory processing and for localizing matching regions across individual subjects that can then be examined more accurately on a group level. The boundaries of repeating, adjacent gradients of one sensory dimension can be determined along the points where the gradient reverses its representation of sensory space (Figure 3). At a gradient reversal, stimulus values represented along the cortical surface increase from low to high (or vice versa) across one CFM to the boundary and then reverse back from high to low (or vice versa) in the next CFM. The boundaries between CFMs are typically drawn to evenly divide the reversals between the two maps, unless additional functional data suggests an alternative approach (e.g., data from a different localizer measurement, like visual motion or face localizers; Press et al., 2001; Brewer et al., 2005; Larsson et al., 2006; Arcaro et al., 2009; Larsson et al., 2010). CFM data are classically visualized as colors overlaid on the cortical sheet that are matched to corresponding stimulus values (Sereno et al., 1995; DeYoe et al., 1996; Engel et al., 1997). Underlying the specific colors are numerical values that are each associated with a specific stimulus, so both manual and automatic approaches to CFM border definitions do not usually rely on just perceived changes in color hue but verify these color changes with the underlying data values (Wandell et al., 2005). If a sensory representation exists in isolation—set apart from other contiguous sensory representations that have already been measured—then the borders may be the edges of the overlapping sensory gradients. In this case, there will likely be some blurring or spreading of the representation along the edges, so special care must be taken in these measurements to not overestimate the extent of the isolated CFM (Engel et al., 1994, 1997; Brewer and Barton, 2012). The definition of the very edge of a CFM therefore may have some inaccuracies, but the affected region should only involve the voxels just along the border. As a result, many studies of sensory CFMs remove the voxels along the border from analyses of the internal CFM organization and functional responses to avoid accidentally incorporate voxels from a neighboring CFM in the analysis (Baseler et al., 2002; Brewer et al., 2005; Baseler et al., 2011; Binda et al., 2013).

The identification of the exact boundaries between CFMs has historically relied mainly on manual determination of the gradient reversals by experts in those specific sensory measurements. When measured by researchers with extensive practice and attention to the stimulus values along the gradient reversal, expert manual definitions have been very reliable across studies in the visual system, the sensory system with the most research devoted to these measurements in the human brain (Sereno et al., 1995; DeYoe et al., 1996; Dougherty et al., 2003; Wandell et al., 2005, 2007). Manual border definitions can also more easily adapt to individual differences in CFM organization and the general “biological noise” observed among CMF measurements (e.g., CFM size differences, map rotations) among individuals (Winawer et al., 2010; Brewer and Barton, 2012; Barton and Brewer, 2017).

The incorporation of more objective approaches for identifying CFM borders is still highly desired, so many groups studying the visual system combine manual border definitions for VFM data with various automated algorithms to aid in their final VFM border determinations (Sereno et al., 1995; Dougherty et al., 2003; Brewer et al., 2005; Larsson and Heeger, 2006). Current algorithms for VFMs are typically applied to both orthogonal eccentricity and polar-angle dimensions simultaneously and often can setup estimations of internal map organization (e.g., iso-eccentricity and iso-angle lines in VFMs). They utilize such approaches as determining the visual-field sign of adjacent VFMs (i.e., mirror vs. non-mirror image representations) or minimizing the error between an expected visual map and the observed data (i.e., atlas-fitting; Sereno et al., 1995; Dougherty et al., 2003; Brewer et al., 2005). The former is best applied to the well-established concentric VFMs of the early visual areas (e.g., V1–V3) and similar maps that abut each other at a polar-angle reversal, while the latter requires careful manual positioning of the initial atlas within the measured data. All the algorithms for VFM definitions that we are aware of to date do best with some level of prior knowledge about the expected pattern of CFM organization, which makes the application of automated algorithms without manual help to the measurement of novel VFMs or new CFMs in the other sensory systems very difficult. This is an area of research ripe for future expansion.

Within the defined CFM boundaries, the orthogonality of the topographical representations should also be assessed, as this is critical to create a topography that uniquely represents sensory feature space. If the topographical gradients for each of these dimensions were parallel instead of perpendicular, the representation of visual space that they would form would be only a spiraling sliver rather than the complete coverage of the visual field that the orthogonal orientation provides (Tyler and Wade, 2005). The orthogonality of the two dimensions can be verified by showing that measurements along the cortical representation of a single value of the first dimension span all values of the second dimension, and vice versa (Figure 1Ci). Each gradient is identified as a series of adjacent vectors which share a trajectory from low to high stimulus values along the cortical surface, though they differ between CFMs in overall size as well as in magnification of ranges of stimulus representation (Baseler et al., 2002; Larsson and Heeger, 2006; Barton et al., 2012; Brewer and Barton, 2016b). Thus, orthogonality can be estimated by measuring the direction of each gradient using a series of vectors drawn either manually or automatically from low-to-high stimulus values and then measuring the angle between the vectors for each gradient (Figure 1Cii; Kolster et al., 2009, 2010; Barton et al., 2012; Brewer and Barton, 2016b; Barton and Brewer, 2017). Due to noise factors within the biological system and measurement limitations of fMRI, some skewing off of 90 degrees can be tolerated, but the two dimensions should still never be parallel (e.g., Larsson and Heeger, 2006). More complex measurements of the fidelity of each gradient and their orthogonality within a CFM have been attempted; for example, researchers in the visual system have used atlas-fitting algorithms to compare the “goodness of fit” of expected representations for a given VFM to the data (Brewer et al., 2005; Dougherty et al., 2005). However, such an approach requires the manual identification of many CFMs in many subjects to have the statistical power necessary, and is not currently feasible for individual CFMs in individual subjects.

### 2.3 Cloverleaf clusters of CFMs

**Figure 4:**
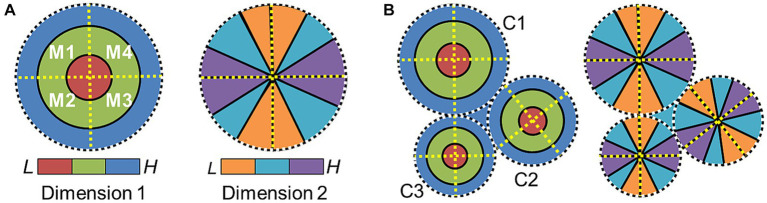
Cloverleaf cluster organization. **(A)** Diagram depicts the representation of one sensory dimension (e.g., eccentricity—vision; tonotopy—auditory) across a flattened region of the cortical surface, with low (red) to medium (green) to high (blue) stimulus values represented in concentric circles (*left*). Diagram depicts the representation of a second sensory dimension (e.g., polar angle—vision; periodotopy—auditory) across the same region of the cortical surface, with low (orange) to medium (cyan) to high (purple) stimulus values represented in wedges running “around the clock” (*right*). Four cortical field maps are defined by these orthogonal gradients and arranged in a cloverleaf cluster (Kolster et al., 2009, 2010; Barton et al., 2012; Brewer and Barton, 2012). Dotted lines denote the boundaries defined by gradient reversals (black/white circle: dimension 1 edge; yellow line: dimension 2 reversal). **(B)** Diagram shows how three cloverleaf clusters each composed of four CFMs can be organized across a region of cortex. Gradient representations for dimension 1 (*left*) and dimension 2 (*right*) are shown on what would be the same overlapping section of the cortical sheet. Dotted black/white circles mark the edge of each cluster (C1–3: Cluster 1–3). Neighboring clusters meet at gradient reversals of high (blue) dimension-1 representations (dotted white/black circles). Boundaries between individual CFMs within each cluster are again marked with dotted yellow lines.

On a larger scale across cortex, CFMs across the majority of human visual and auditory cortex studied to date are organized into patterns called cloverleaf clusters (Brewer et al., 2005; Wandell et al., 2005, 2007; Kolster et al., 2010; Barton et al., 2012; Brewer and Barton, 2012; Barton and Brewer, 2017). This macrostructural pattern has also been observed in the visual system of the macaque (Kolster et al., 2009). Figure 4A depicts the arrangement of the two topographical gradients that compose the CFMs of a single cloverleaf cluster. The “cloverleaf” term comes from the pattern of CFMs arranged within a cloverleaf cluster like the leaves of a clover plant. Dimension 1 is organized such the sensory topography moves from low to high along concentric, circular bands (e.g., visual eccentricity and auditory tonotopy), with an orthogonal dimension 2 then arranged as repeating gradients running in radial bands from the center to the periphery of the representation of dimension 1 like spokes on a wheel (e.g., visual polar angle and auditory periodotopy; Brewer et al., 2005; Wandell et al., 2007; Barton et al., 2012; Brewer and Barton, 2016b). Reversals in dimension 2 divide a single cluster into individual CFMs, while reversals in dimension 1 serve as boundaries among cloverleaf clusters (Figure 4B). This macrostructural pattern is now described as being radially orthogonal (Brewer and Barton, 2012).

The spatial organization of cloverleaf clusters is reminiscent of the organization of orientation pinwheels at a smaller spatial scale, with both consisting of smoothly changing representations that appear to blend together across swaths of cortex (Grinvald et al., 1986; Bonhoeffer and Grinvald, 1991; Maldonado et al., 1997; Ohki et al., 2006). Grouping together neurons with similar selectivity in this way is likely to not only help minimize axonal connectivity to optimize energetic efficiency, but also to influence synaptic integration and coordinate neural computations (Schummers et al., 2002; Chklovskii and Koulakov, 2004; Shapley et al., 2007; Moradi and Heeger, 2009). It is thus thought that neurons within each cluster share common computational resources, such as short-term information storage, or coordinate neural timing across the sensory hierarchy (Press et al., 2001; Brewer et al., 2005; Wandell et al., 2005; Barton et al., 2012; Barton and Brewer, 2017; Landi et al., 2021; Qasim et al., 2021). Perceptual specializations, such as visual processing of color or motion, similarly appear to be mostly organized by clusters of CFMs rather than individual CFMs (Zeki and Bartels, 1999; Bartels and Zeki, 2000; Brewer et al., 2005; Brewer and Barton, 2018). The MT cluster with homologous organizations in human (TO or hMT^+^) and macaque (MT^+^) is an excellent illustration of this cluster-based perceptual processing (Wandell et al., 2007; Amano et al., 2009; Kolster et al., 2009, 2010). The MT^+^ cluster in macaque is composed of four VFMs—MT, MST, FST, and V4t, all of which contribute to unique stages of visual motion perception (Kolster et al., 2009). The hMT^+^ cluster in human similarly contains 4 VFMs involved in visual motion, although the specific homologies to the macaque VFMs are still under study (Huk et al., 2002; Kolster et al., 2010; Brewer and Barton, 2012). Such cloverleaf cluster organization of CFMs likely reflects how multiple stages in a sensory processing pathway might arise through evolution.

It will be interesting for future research to determine how widespread the cloverleaf cluster organization is across the senses. VFMs in the frontal lobe such as the frontal eye fields (FEF) and the regions in the dorsolateral prefrontal cortex (DLPFC) appear in currently published data to be isolated retinotopic hemifield representations that are not organized into cloverleaf clusters, but there are emerging reports from preliminary data that additional maps are present in these regions as well that may be organized into clusters (Hagler and Sereno, 2006; Saygin and Sereno, 2008; Silver and Kastner, 2009). The small number of AFMs that have been measured in human auditory core and belt do appear to be organized into cloverleaf clusters, but we know little yet of the topographical representations of auditory dimensions that likely extend along the lateral fissure (Barton et al., 2012; Brewer and Barton, 2016b). While cloverleaf clusters have not yet been observed in our current measurements of the somatosensory system or the chemical senses, we have only limited measurements of the associated topographical representations for each in the human brain. A more complete understanding of the extent of cloverleaf-cluster organization will be important for insight into how such topographical representations evolved across the senses and among species (Krubitzer, 2007; Wandell and Smirnakis, 2009).

### 2.4 CFM comparisons across cortex and species

As our study of CFMs expands across cortex and species, it is useful to keep in mind the possible ways that these representations may be changing under evolutionary pressures. Evolution is ongoing, continually molding organisms as their environments change. The organization and functional specialization of CFMs and cloverleaf clusters are unlikely to have reached an evolutionary endpoint, so the cortical sensory representations that we are measuring may not be perfectly organized or may show specific types of variations across individuals or species (for detailed discussion, see Krubitzer, 2007). Consideration of these types of changes can help to improve our localization of specific CFMs across individuals, our identification of new CFMs in various sensory systems, and our recognition of the homologies across species.

**Figure 5:**
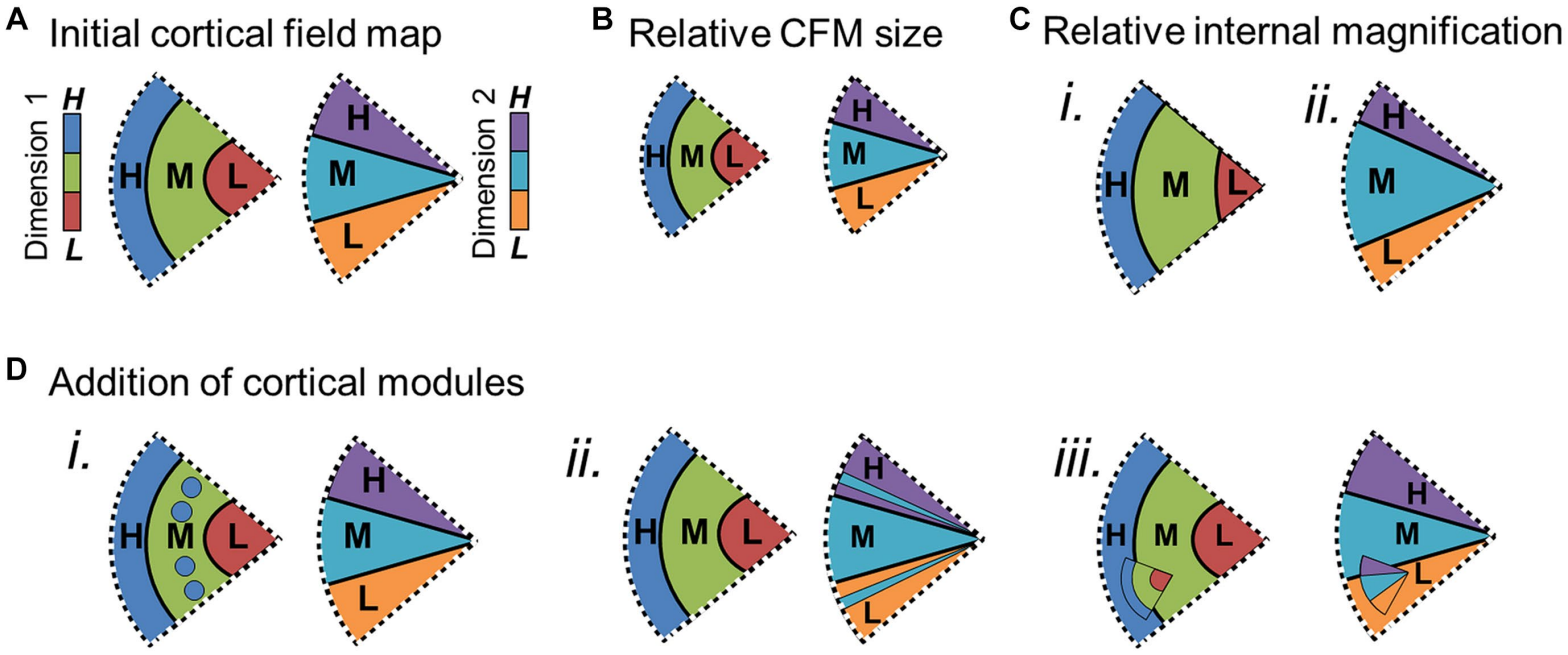
Potential changes within cortical field maps over evolution. Schematic diagrams depict several ways cortical field maps can change over the course of evolution, important for consideration of potential homology of CFMs among species, individuals, and sensory cortices (see Krubitzer and Seelke, 2012 for extended discussion). Each schematic shows two pictures of the same CFM, one for each orthogonal dimension (e.g., dimension 1: visual eccentricity; dimension 2: visual polar angle). **(A)** Example of the baseline CFM with 3 colors coded for representations of the low (L), middle (M), and high (H) sensory values for each orthogonal dimension. Subsequent schematics show changes with respect to this initial CFM. **(B)** Overall size of CFM may be reduced. **(C)** The magnification of a particular part of the internal representations [e.g., middle value (M)] may increase for dimension 1 **(i)** and/or dimension 2 **(ii)**. **(D)** New representations may be in the process of emerging or combining within a complete CFM. **(i)** Additional segments of high-value (H; blue) representations of dimension 1 are present within the medium-value (M; green) representations. **(ii)** Additional segments of medium-value (M; cyan) representations of dimension 2 are present within the high-value (H; purple) and low-value (L; orange) representations. **(iii)** A smaller complete CFM exists within the larger CFM. Other details are as in Figure 4.

**Figure 6:**
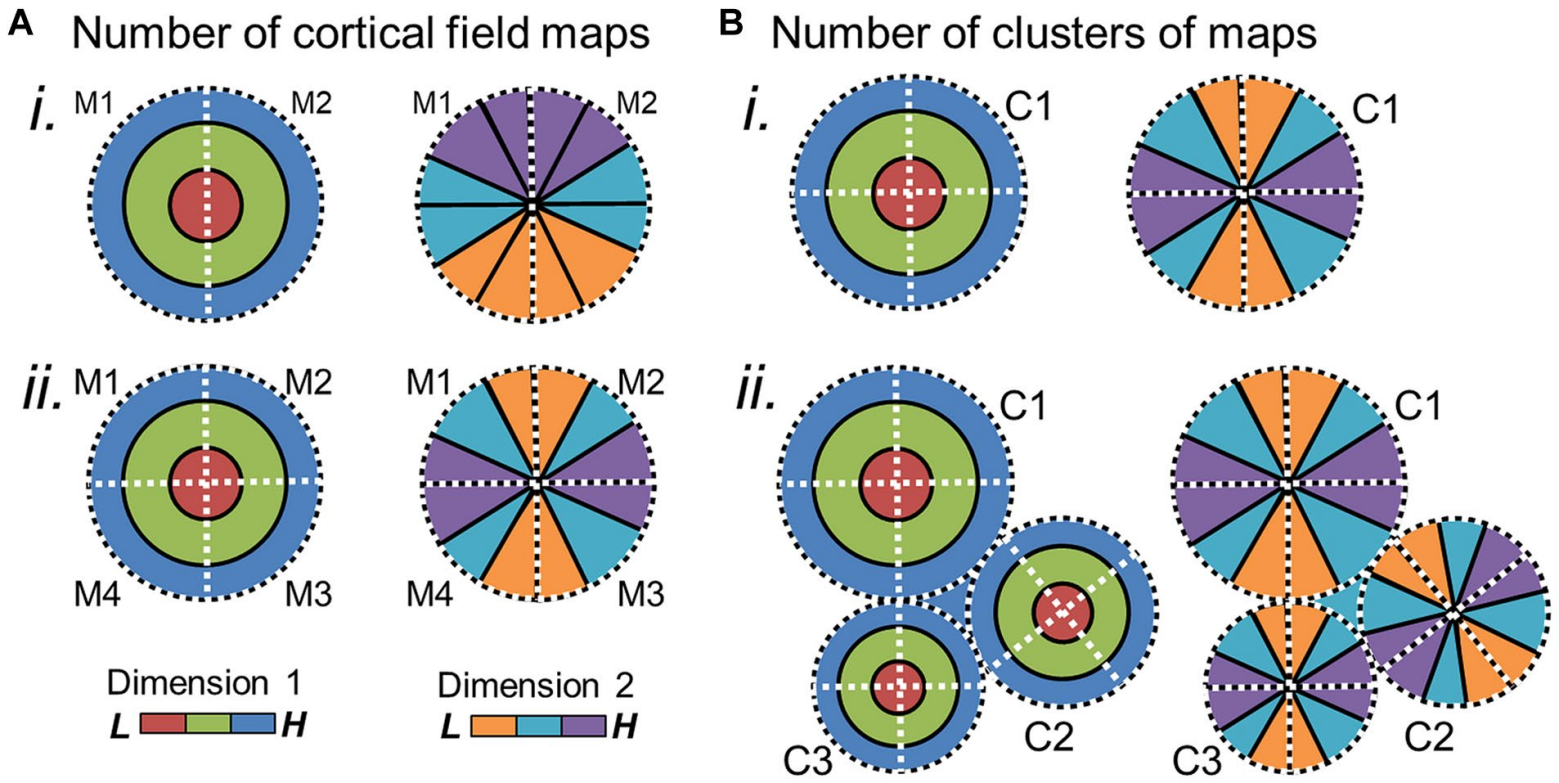
Potential changes in cloverleaf clusters over evolution. **(A) (i)** Schematic depicts the two orthogonal gradients for a cloverleaf cluster composed of two CFMs. **(ii)** Evolution of additional CFMs within the cluster could alter the internal structure of the cluster to now be composed of 4 CFMs. **(B)** New clusters of CFMs may emerge in adjoining regions, with the cortical-sheet territory around one cluster **(i)** expanding to include more distinct clusters **(ii)**. Such expansions of CFMs within a cluster or of clusters themselves may correlate with expansions in the related sensory behaviors. M1–4 = map 1–4; C1–4 = cluster 1–4. Other details are as in Figures 4, 5.

**Figure 7:**
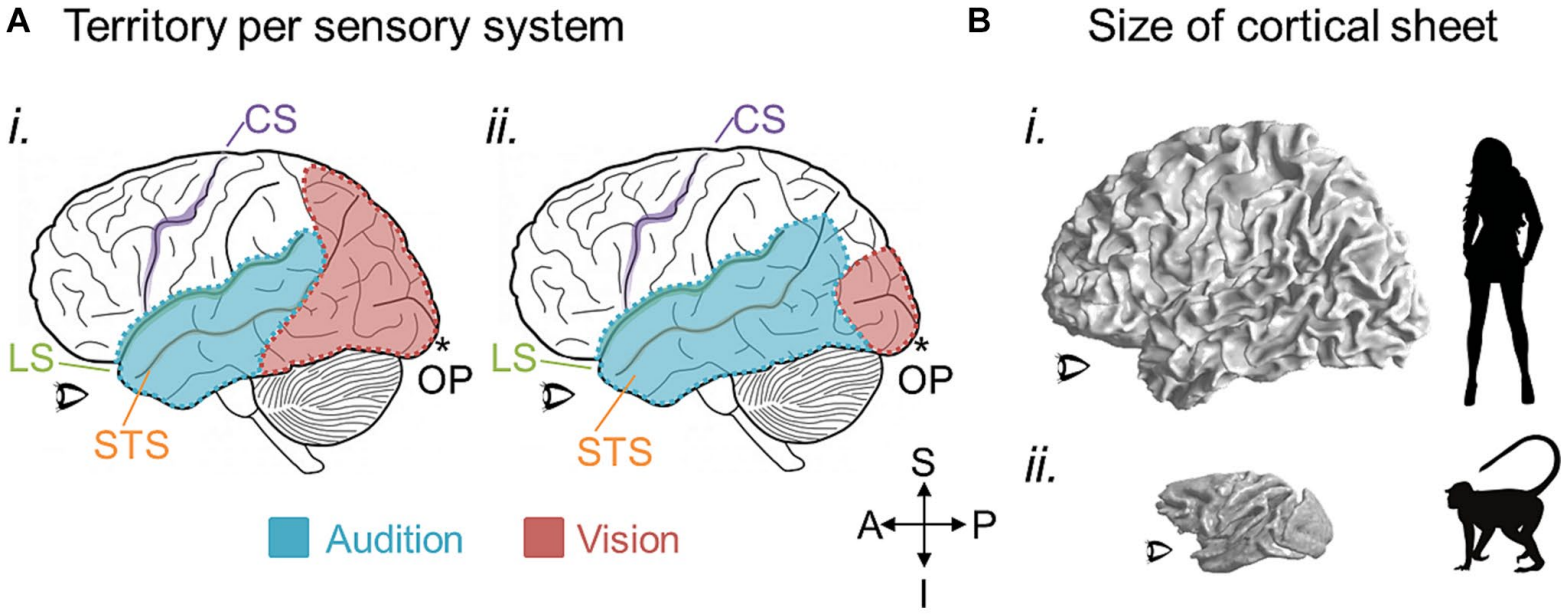
Potential large-scale brain changes over evolution. **(A)** The amount of cortical territory devoted to one sensory system may cede territory to another in conjunction with the expansion or reduction of their related behaviors. Cartoons of a left hemisphere are shown with colored overlays representing hypothetical regions of cortex devoted to auditory processing (blue) and visual processing (red). In **(i)**, more cortex is devoted to visual than auditory processing, while the opposite would be true in **(ii)**. CS, central sulcus (purple); LS, lateral sulcus (green); STS, superior temporal sulcus (orange); OP, occipital pole (*). **(B)** 3-D inflated renderings of a human brain **(i)** and macaque brain **(ii)** are scaled for approximate relative size and shown at the white-gray boundary to demonstrate the changes in the size of the cortical sheet over 25 million years of evolution between the two species (Hedges and Kumar, 2003). An increase in overall cortical sheet size could accommodate expansions of CFMs, such as those depicted in Figures 5, 6, and lead to correlated increases in the complexity of associated behaviors. Anatomical-directions legend: S, superior; I, inferior; P, posterior; A, anterior.

Figures 5–7 demonstrate several changes that CFMs may be undergoing across individuals, species or sensory domains (Krubitzer and Seelke, 2012). The overall size of a CFM may vary across individuals or species (Figures 5A,B), or there may be changes in the cortical magnification of specific parts of the internal topography of a CFM that correlate with differences in sensory experiences or perceptual needs (Figure 5C). In the human visual system, for example, primary visual cortex (V1) can vary by at least a factor of three in surface area, independent of overall brain size (Dougherty et al., 2003). Research is still exploring how these differences in V1 size correlate with differences in visual behavior and sensory sensitivity. Along these lines, Schwarzkopf and Rees (2013) found that illusory size perception can be influenced by differences in the cortical magnification of the central foveal representation in V1. More complex changes in internal topography can also arise among individuals or species, such as the emergence of small modules or sub-maps within a section of a CFM (Figure 5D). The appearance of such sub-topographies may reflect adaptations driven by early developmental differences or experience in particular individuals or may be the result of mutations that could eventually lead to the emergence of new cortical maps within a particular sensory system.

On a larger scale, there may be changes in the numbers of CFMs and/or their cloverleaf clusters across a region of the cortical sheet or devoted to a particular perceptual processing pathway (Figure 6). An expansion in the number of CFMs may underlie an expansion in perceptual abilities, with new CFMs supporting new aspects of behavior. For example, visual object recognition in human arises from a large swath of cortex that contains numerous VFMs that are thought to support various aspects of visual object processing: e.g., hV4; VO-1, VO-2; PHC-1, PHC-2; LO-1, LO-2 (Wade et al., 2002; Brewer et al., 2005; Wandell et al., 2005; Larsson and Heeger, 2006; Larsson et al., 2006; Montaser-Kouhsari et al., 2007; Arcaro et al., 2009). In comparison, the homologous regions in macaque monkey are relatively much smaller, comprising such areas as TEO and V4 (Desimone and Schein, 1987; Gattass et al., 1988; Boussaoud et al., 1991; Tanaka et al., 1991; Nakamura et al., 1993; Brewer et al., 2002). With ~25 million years of evolution separating humans and macaques, it is not surprising that we see differences in the complexity of visual object processing that are likely associated with the similar differences in the complexity of object use (Hedges and Kumar, 2003; for additional discussion, see DiCarlo et al., 2012). One can imagine genetic duplications reminiscent of the homeobox genes involved in body-structure patterning or the eph/ephrin pairs driving topographical connectivity that could underlie the expansion of cloverleaf clusters of CFMs and their associated behaviors; additional CFM clusters may emerge through genetic duplications and thus provide an increase in cortical territory available to support a more complex range of behaviors in a particular sensory processing stream (Crawford, 2003; Kmita and Duboule, 2003; Holland and Takahashi, 2005; Lappin et al., 2006). Such expansions in cortical territory and associated behavioral complexity are indeed observed across sensory systems and among many species (Figure 7; Krubitzer, 2007; Krubitzer and Seelke, 2012).

## 3 Measurement techniques for cortical field maps

### 3.1 Phase-encoded fMRI: using traveling-wave stimulation to measure CFMs

**Figure 8:**
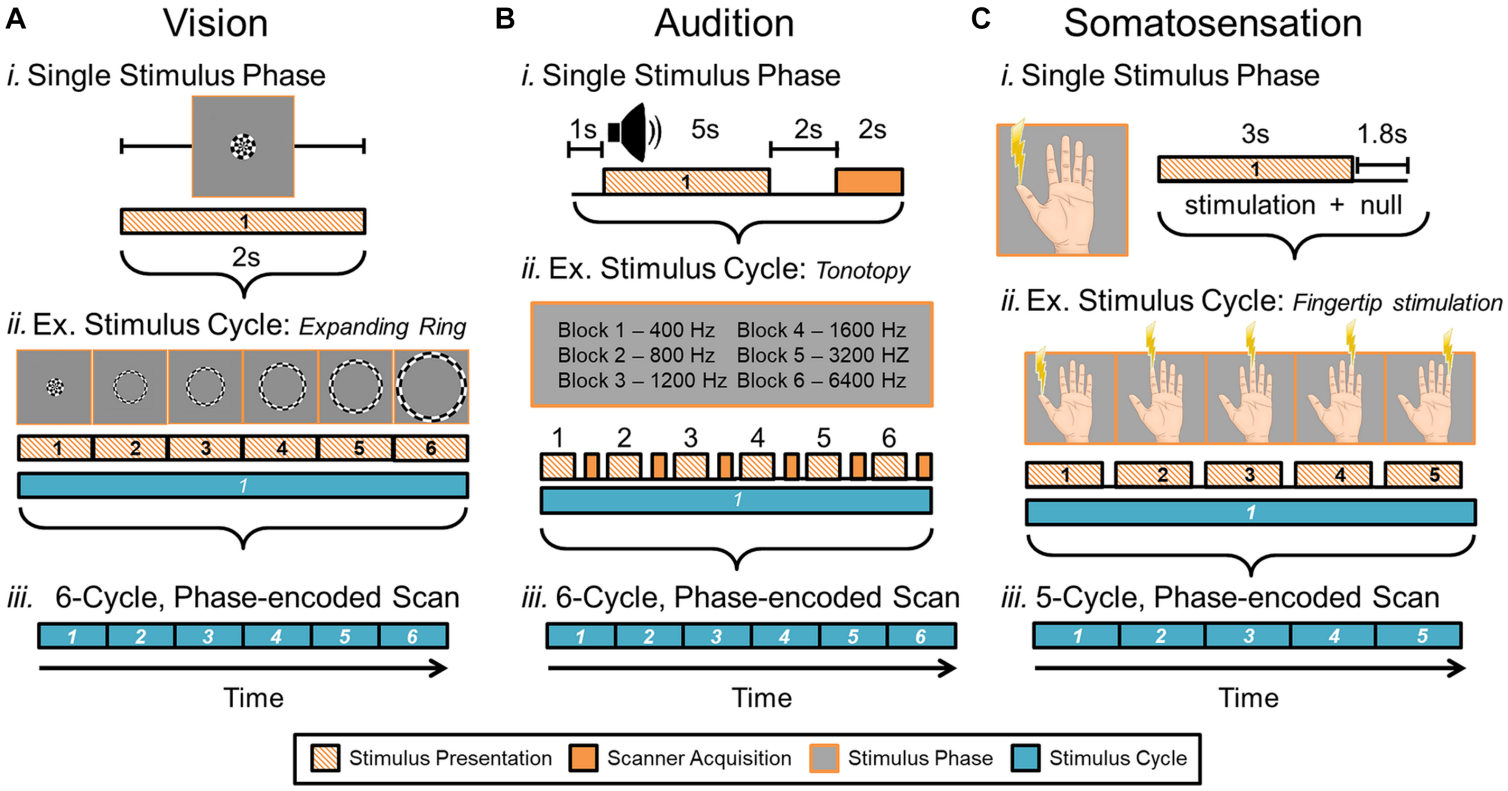
Phase-encoded neuroimaging paradigms for cortical field mapping. (A) Schematic presents an example paradigm for measuring visual field maps using phase-encoded fMRI. **(i)** Typical stimuli used for visual field mapping are composed of black and white moving checkerboard patterns on a neutral grey background, as show for the expanding ring stimulus. A 2-second presentation of this example visual stimulus, stimulating one central position in visual space (i.e., one stimulus phase) is represented by the striped orange bar. For visual stimuli, scanner acquisition occurs simultaneously with the stimulus presentation (Engel et al., 1994; Wandell et al., 2007; Brewer and Barton, 2012). **(ii)** One full stimulus cycle consists of several blocks of the visual stimulus stepping through visual space. Each phase of the expanding ring stimulus is displayed above the blocks; one block thus represents one stimulus position in the ‘phase-encoded’ sequence. The six striped-orange blocks together compose one stimulus cycle (cyan bar). The term ‘travelling wave’ is also used to describe this type of stimulus presentation, as the stimuli produce a sequential activation of representations across a topographically organized cortical region. **(iii)** A full, single scan to measure VFMs is then composed of a number of cycles of the stimulus moving through visual space (e.g., 6 cycles shown in cyan). **(B)** Schematic presents an example paradigm for measuring auditory field maps using phase-encoded fMRI. **(i)** The top diagram shows how the auditory stimulus presentation (striped orange bar) is separated from the noise of the scanner acquisition (solid orange bar) in a phase-encoded, sparse-sampling fMRI paradigm (Petkov et al., 2009). The delayed timing of the acquisition collects the peak cortical response to the auditory stimulus, in accordance with the approximate hemodynamic delay. **(ii)** Typical stimuli used for auditory field mapping consist of a series of tones, frequencies, or noise bands (e.g., narrow-band noise for tonotopy and broad-band noise for periodotopy), as shown in the gray table. Each stimulus block is composed of a single tone or noise band and the scanner acquisition period. The diagram shows 6 blocks (striped orange + solid orange) of consecutive frequency ranges grouped together into one stimulus cycle (cyan bar). **(iii)** The diagram again shows a full, single scan comprising 6 cycles. **(C)** Schematic presents an example paradigm for measuring somatosensory field maps using phase-encoded fMRI. **(i)** Stimuli used for somatosensory field mapping of the fingertips to date have been composed of sequential stimulation of the fingertips by piezo-electric stimulators for vibrotactile sensation, air puffs for light touch, or radiant-heat lasers for pain. A stimulus block consists of the stimulation (orange striped bar) and a null period. **(ii)** Phase-encoded measurements again step through the sensory space (e.g., each fingertip) over one stimulus cycle (cyan bar). **(iii)** As for the other sensory modalities, a single scan consists of multiple cycles, e.g., five in this example. Note color legend in inset.

One of the gold standards for measuring CFMs in human using fMRI is a phase-encoded paradigm that relies on a stimulus sequentially activating regions across sensory space (Figure 8; Engel et al., 1994; Sereno et al., 1995; DeYoe et al., 1996; Sanchez-Panchuelo et al., 2010; Barton et al., 2012; Mancini et al., 2012; Kolasinski et al., 2016a). “Phase-encoded” refers to the tie between the cortical activation and the periodic sensory stimulus; as the stimulus moves through sensory space, neural activity increases within the corresponding cortical sensory representations. With repetitions of the stimulus movement, the neural activity within the associated cortical representations is modulated in sync with the stimulus repetition. The cortical response is matched to its sensory topography through its correlation to the timing, or phase, of the stimulus presentation.

To measure one sensory dimension with this paradigm, like eccentricity in vision or tonotopy in audition, a set of stimulus values is presented in an orderly sequence across a range of interest. In the visual system, commonly used stimuli include expanding rings and rotating wedges that are used to measure the dimensions of visual eccentricity and polar angle, respectively. Such retinotopic stimuli are typically composed of a moving checkerboard pattern, which is designed to maximize the response of primary visual cortex (V1; Engel et al., 1994; Sereno et al., 1995; DeYoe et al., 1996; Wandell et al., 2007). To measure visual eccentricity, for example, the expanding ring stimulus would start as a small disc in the center of the field of view (i.e., at the fixation point) and would sequentially step out as a narrow annulus from the center out to the visual periphery (Engel et al., 1997). This range would constitute one stimulus cycle (Figure 8A). Over a single scan, the expanding ring would repeat this movement several times to increase the power of the measurement. For tonotopic measurements, a set of frequencies would be presented in order from low to high for one stimulus cycle, for example, and this cycle would again repeat several times during one scan (Figure 8B; Talavage et al., 2004; Humphries et al., 2010; Barton et al., 2012; Brewer and Barton, 2016b). With auditory stimuli, the MR scanner noise must be taken into account as a possible source of contamination of the auditory signal. A sparse-sampling approach separates the auditory stimulus from the scanner acquisition noise by separating the two in time (Figure 8Bi; Bandettini et al., 1998; Scarff et al., 2004; Gaab et al., 2007; Petkov et al., 2009; Joly et al., 2014). Somatotopic measurements ideally would similarly arise from sequential activation over the entire skin or dermal zone of interest. Due to the complexity of such stimulation across such a large organ as the skin and within the MR environment, somatotopic tactile and pain measurements have so far been restricted to more selective sampling across a relatively restricted region, such as the fingertips or selected points across the body on the head, finger/hand, body, and foot/leg (Figure 8C; Ruben et al., 2001; Sanchez-Panchuelo et al., 2010; Mancini et al., 2012; Kolasinski et al., 2016a; Sanchez Panchuelo et al., 2018; Schellekens et al., 2018; Willoughby et al., 2020). For all of these approaches, the value of the stimulus that most effectively drives each cortical location—e.g., specific degrees of visual eccentricity, auditory frequency, or location on the skin—is then estimated from the pattern of neural responses.

**Figure 9:**
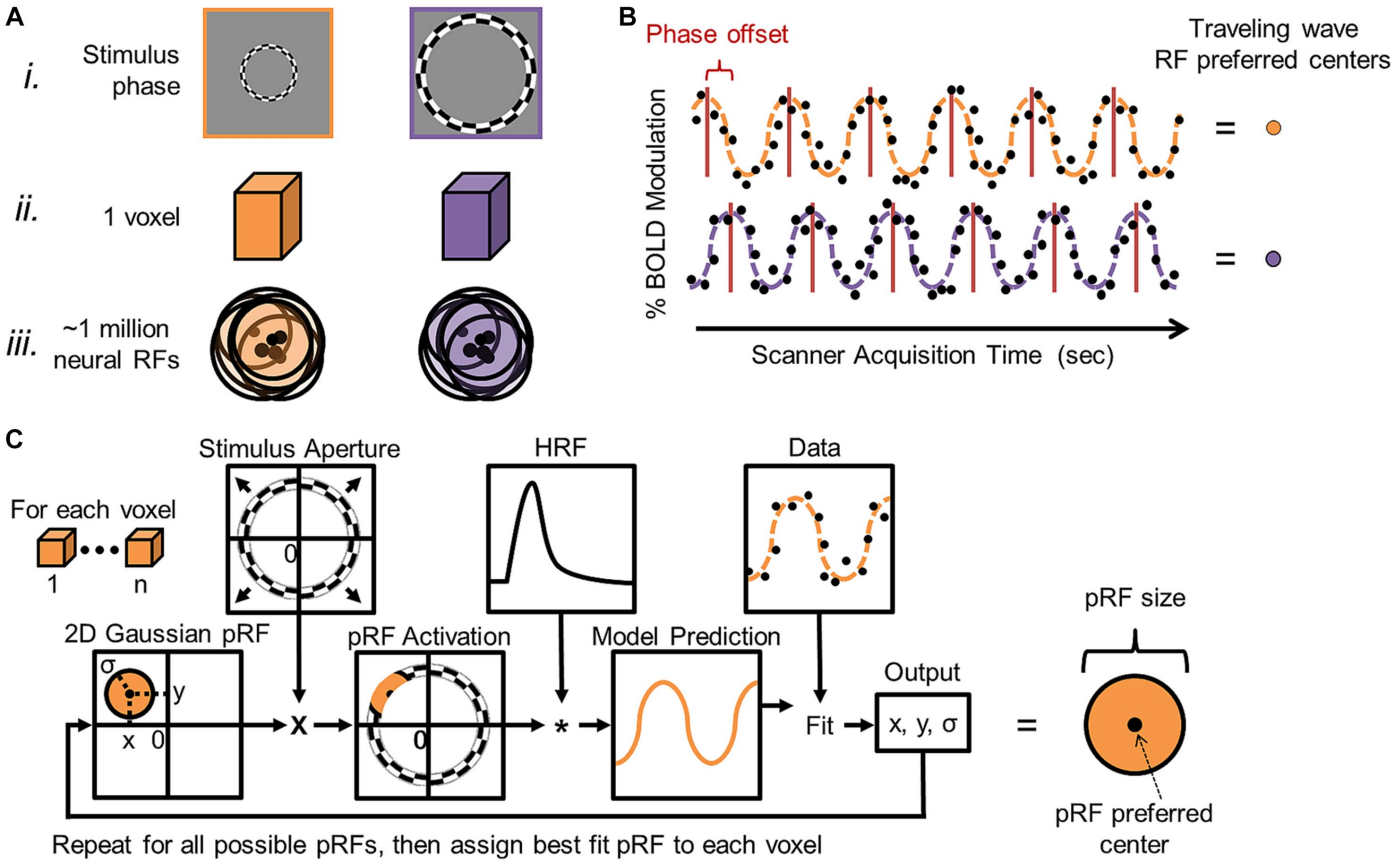
Cortical field mapping analysis. **(A) (i)** Schematic measurements are shown for two different phases of an expanding-ring visual stimulus (orange = earlier phase; purple = later phase). Although there are on the order of ~1 million neurons within a typical voxel **(ii)** measured with 3 T MRI for cortical field mapping (Hoffmann et al., 2007; Brewer and Barton, 2016b), such neighboring neurons in topographically organized sensory cortex each have similarly tuned receptive fields **(iii)** (orange and purple circles with black outlines) with similar preferred centers of maximal response (black dots). Note how the overlapping receptive fields concentrate coverage in one region of sensory space corresponding to the average receptive field of the group. Phase-encoded measurements rely on this organization to estimate the average preferred center for the population of neurons in a given voxel. **(B)** Diagram displays two example phase-encoded time series with different stimulus responses arising from the orange and purple visual stimuli, respectively. Each plot shows the time series of a single 6-cycle scan of one type of experimental stimuli (e.g., expanding rings) for a single voxel. Note that, in phase-encoded paradigms, only BOLD responses that match the stimulus frequency in terms of cycles per scan are considered as data (Engel et al., 1994; Dumoulin and Wandell, 2008; Brewer and Barton, 2012). Simulated raw data points of percent blood-oxygen-level-dependent (BOLD) modulation (i.e., response amplitude) are indicated by the black dots, while the orange and purple dotted lines denote the sinusoidal fits for two example simulated datasets. Red lines indicate the peak activations per stimulus cycle for these two simulated voxel activations. The horizontal offset of the red lines between the orange and purple sinusoids indicates differences in stimulus selectivity for the populations of neurons in each voxel, as each example voxel is responding to a different stimulus phase. These activations that are encoded to the phase (timing/position) of the stimulus (hence, the term phase-encoded fMRI paradigm) are then represented by different colors in the pseudocolor overlays representing cortical field maps (see VFM schematic shown in Figure 10). Adapted from Brewer and Barton (2012). **(C)** Population receptive field (pRF) modeling was developed for visual field mapping in order improve measurements in higher-order visual cortex. This additional analysis allows for not only the measurement of the peak activation (i.e., preferred RF center) for a particular voxel as described in **(A,B)**, but also for the measurement of the average pRF size for the population of neurons in a given voxel (Dumoulin and Wandell, 2008). The parameter estimation procedure for the pRF model is shown as a flow chart. PRF modeling has now also been adapted for measuring tonotopic gradients (Saenz and Langers, 2014; Thomas et al., 2015; Lage-Castellanos et al., 2023) and discrete somatotopic and motor topographical locations (Schellekens et al., 2018). Based on Figure 2 in Dumoulin and Wandell (2008).

With this phase-encoded experimental paradigm, only cortical regions that show a modulation of activity in sync with the stimulus modulation are included in the CFM analysis (Figures 9A,B; for extended discussions, see Wandell et al., 2005; Brewer and Barton, 2016b). Regions that are active at other, non-stimulus frequencies are not included in the measurement. So, for example, if a region responds to the presence of any visual stimulus anywhere in the visual field, that region will remain active throughout the visual stimulus presentation, rather than being active only when the stimulus moves through its spatially restricted zone in the visual field. Only those regions organized around a sensory topography will show phase-encoded activity in response to the traveling-wave stimulus.

### 3.2 Specialized approaches: population receptive field modeling

A more specialized, model-based approach has been developed to measure VFMs in human cortex using a range of visual stimuli that periodically move through visual space, including the traditional traveling-wave/phase-encoded measurements. This method can collect additional information about VFMs by modeling the population receptive field (pRF) of each voxel within a VFM (Figure 9C; for complete pRF-modeling details, see Dumoulin and Wandell, 2008; for examples of pRF-modeling applications, see Baseler et al., 2011; Haak et al., 2012; Barton and Brewer, 2015, 2017). Within an organized sensory topography, receptive fields (RFs) in each small voxel typically have such similar representations of visual space that the combined, average RF across the population of neurons within each voxel can be estimated as a single, 2D Gaussian. The pRF-modeling method thus provides an assessment of not only the preferred center for the pRF of each voxel, as is measured with phase-encoded mapping alone, but also its size. Although there is some variability in the neural RFs of each voxel in terms of their preferred centers and sizes, termed RF scatter, the pRF analysis provides a good, if somewhat slightly larger, estimate of the individual visual RFs in the voxel. Research is currently underway to develop similar pRF models for auditory and somatosensory field maps. To date, pRF modeling has been adapted to measuring tonotopic responses—one dimension of AFMs—in human primary auditory cortex (Saenz and Fine, 2010; Thomas et al., 2015; Lage-Castellanos et al., 2023) as well as modeling somatotopic and motor responses at discrete points across the fingers and body (Schellekens et al., 2018).

### 3.3 Considerations for data acquisition and analysis of CFM measurements

Obtaining a high-quality measurement of topographic cortical representations is a vital step in the accurate definition of CFMs and relies in part on the selection of appropriate phase-encoded stimuli. First, the sampling density of the stimulus values across sensory feature space heavily influences the precision of the CFM measurement. If, for example, a visual stimulus only activates the far periphery of the visual field, then the resulting VFM measurement will be skewed from the actual map (Wandell et al., 2005, 2007). While such a restricted field of view is a rather unlikely approach for visual field mapping, the issue becomes much more of a pressing problem when we do not have as clear an understanding of the sensory feature space as we do for visual space. If stimulus values are chosen that are not included within the topographic gradient in a particular CFM, then the attempted measurements will fail to reveal an organized topographical representation within the purported area of the CFM. Similarly, if the sampling density of the stimulus values is too coarse, the precision of the CFM measurements will be poor, because the gradients can only be estimated from the interpolation of just a few sampled responses. When only a few stimulus values are tested across a wide range of sensory space, e.g., only 0° and 90° of visual angle or 400 Hz and 64,000 Hz for auditory stimulation, many parts of the associated cortical representations will be only weakly activated, because no stimulus falls within their preferred stimulus selectivity (Barton et al., 2012; Brewer and Barton, 2016a). Consequently, the fMRI measurements at those cortical locations will be inaccurate, as they would be determined mainly by signals that spread from activity in the surrounding cortex that contains neurons with different stimulus preferences. The estimated stimulus preference for these regions will also be contingent to a much greater degree on the spatial spread of the blood-oxygenation-level-dependent (BOLD) signal that underlies the fMRI measurement (Engel et al., 1994, 1997). This spreading process basically blurs the data and is subject to other, variable characteristics of the brain, such as vascular density, that can add additional noise into the CFM measurements at that cortical location (for review, see Logothetis and Wandell, 2004; Winawer et al., 2010).

Similar issues can arise from distortions, signal dropouts, and other artifacts in the fMRI data that can be introduced through interactions between these non-invasive BOLD measurements and the adjacent anatomy and tissues of the head and neck (Logothetis, 2002; Logothetis and Wandell, 2004; Yu et al., 2023). For example, definitions of VFMs in the ventral visual pathway were controversial for many years due to inconsistencies in the measurements across individual subjects until it was shown that differences in the vascular pattern in the region could cause a venous eclipse in the data that erased the measurement of certain ventral VFMs in some subjects (e.g., hV4 Brewer et al., 2005; Winawer et al., 2010). In an ideal world, researchers could compare an image of the vascular system and BOLD data for each individual brain to account for such signal loss, but that is not currently feasible. Larger-scale structures such as dural venous sinuses and air cavities have hindered data collection of certain sensory data across the majority of subjects, leading to the general misinterpretation of sensory processing in the adjacent cortical regions (Zeki, 2003; Brewer et al., 2005; Du et al., 2007; Wandell et al., 2007). Measurements of human auditory cortex are far behind those of visual cortex in part due to distortions and signal loss introduced by the air pockets of the ear canal into fMRI data collected along the lateral fissure (Peelle, 2014; Talavage et al., 2014; Brewer and Barton, 2016b). Improvements in acquisition protocols and increased spatial resolution have now helped measurements overcome this issue for the most part. Regions near orbitofrontal cortex similarly are affected by signal loss caused by neighboring air cavities, thus limiting the measurements of higher-order regions of olfactory and gustatory processing (Hutton et al., 2002; Zelano and Sobel, 2005; Du et al., 2007). The brain anatomy itself can produce limits on the spatial resolution that can be obtained in certain regions, such as the closely abutting gyri of primary somatosensory cortex (S1) and primary motor cortex (M1) across the central sulcus (Penfield and Boldrey, 1937; Woolsey et al., 1979). Partial-voluming effects from single voxels combining data from gray matter on both sides of the sulcus has led to a comparable delay in our ability to properly define human S1 and M1 topographic organization with fMRI (Gonzalez Ballester et al., 2002; Duncan and Boynton, 2007; Besle et al., 2013; Sánchez-Panchuelo et al., 2014; Schellekens et al., 2018; Willoughby et al., 2020). The majority of these artifacts also differ across magnetic field strength, adding an additional layer of complexity (Maldjian et al., 1999; Benson et al., 2018; Morgan and Schwarzkopf, 2019). Choice of the appropriate distortion correction during MRI data collection and post-acquisition processing is therefore invaluable for these measurements.

Specific analysis approaches can also affect the ability to measure CFMs across sensory systems. Methods that reduce the spatial resolution are particularly prone to destroying or altering the topographical measurements composing a CFM. For example, smoothing phase-encoded measurements with a Gaussian kernel can destroy important internal topographical features within a larger CFM or miss a smaller CFM entirely (Brewer et al., 2005; Winawer et al., 2010). Issues with anatomical image analysis can also similarly obliterate CFM measurements. Segmentation of white matter from gray, commonly needed for individual-subject data analysis in particular, requires not only high-quality automated segmentation algorithms, but also careful researcher review and hand-editing to ensure that the cortical sheet is properly defined, especially along the peaks of the gyri and the depths of the sulci (Nestares and Heeger, 2000; Brewer et al., 2002). Otherwise, the topographical data will be inappropriately missing regions that fall at these anatomical regions or blurring regions together across two gyri, such as early measurements of primary somatosensory (S1) and motor (M1) cortices that often blended together responses within single voxels crossing the central sulcus (i.e., partial-volume effects; for discussion, see Gonzalez Ballester et al., 2002; Wandell et al., 2007; Sanchez-Panchuelo et al., 2010). The measurement of a CFM is thus much more significant than the failure to find a map, especially when a particular CFM is reliably found across most observers (Brewer et al., 2005; Wandell et al., 2005, 2007; Winawer et al., 2010; Brewer and Barton, 2012).

Furthermore, the accurate definition of CFM boundaries relies on the analysis of sensory measurements from individual subjects. Averaging topographical measurements across a group, especially by aligning the data to an average brain through such atlases as Talairach space (Talairach and Tournoux, 1988) or Montreal Neurological Institute (MNI) coordinates (Collins et al., 1994), typically introduces significant blurring into the data (Brewer et al., 2005; Wandell et al., 2005). The relationship between cortical anatomy and CFM functional responses is variable enough across individuals, that such group-averaging is likely to misalign the appropriate topographies with other CFMs or unrelated cortical regions (Dougherty et al., 2003). As a result, the gradients composing the CFMs may be inaccurate or even missing (Sereno et al., 1995; DeYoe et al., 1996; Engel et al., 1997; Wandell et al., 2007; Barton et al., 2012; Brewer and Barton, 2012; Baumann et al., 2015). As we expand CFM measurements across the senses, such factors need to be taken into careful consideration.

## 4 Topographical representations in human sensory cortex

Over the last century, extensive research has been dedicated to unraveling the intricate mechanisms that underlie sensory perception and their associated cortical topographies. Within the visual, auditory, and somatosensory systems, researchers have made significant strides in understanding how specialized receptors in peripheral sense organs transduce and analyze crucial physical properties of external stimuli and ultimately how this sensory information is organized across sensory cortex (e.g., Wandell et al., 2007; Barton et al., 2012; Brewer and Barton, 2012, 2016a; Sanchez Panchuelo et al., 2018; Willoughby et al., 2020). We can now reliably measure a number cortical field maps or organized topographies within these systems, as described in the following sections.

In contrast, the chemical senses of taste and olfaction present unique challenges when it comes to representing stimulus features in the brain (Imai et al., 2010; Murthy, 2011). Unlike measurable dimensions such as the spatial positions across the visual field and skin surface or the spatiotemporal frequencies within sound waves, which all more naturally lend themselves to spatial organization in the cortex, the molecules relevant to the chemical sense organs do not possess such continuous physical properties, except for their magnitude or intensity (Chaudhari and Roper, 2010; Ma et al., 2012). Instead, the quality of a chemical stimulus is determined by its chemical composition, which lacks variation along a common physical dimension across different substances. As a result, our understanding of the cortical representations of smell and taste remains substantially more limited than that of the other senses. Even so, organized topographies within the chemical senses are emerging as well (Chen et al., 2011, 2021, 2022; Prinster et al., 2017; Lodovichi, 2020).

### 4.1 Visual field maps

The spatial arrangement of a visual image is a critical aspect of our ability to recognize elements of our environments (Sereno et al., 1995; DeYoe et al., 1996; Engel et al., 1997; Wandell et al., 2005, 2007). While an image may still be identifiable despite alterations of such properties as its color, motion, contrast, or rotation, scrambling its spatial arrangement typically destroys our ability to identify or reconstruct the original image. This visual field spatial arrangement is encoded by the circuitry of the retina and then preserved and repeated through visual cortex to produce a unifying matrix of visuospatial organization throughout the visual processing hierarchy, despite the diverse computations being performed across regions (e.g., Van Essen, 2003; Wandell et al., 2007; Brewer and Barton, 2012). As cortex interprets different aspects of the visual image—such as its motion or orientation—the cortical circuitry is organized using receptive fields arranged within VFMs to preserve the critical spatial image information.

In lower-level VFMs, precise measurements are taken of low-level visual features in a particular retinal location, which are built up into more complicated localized representations as they are processed through the cortical hierarchy. Despite having large receptive fields, higher-order visual cortex may still maintain visuospatial organization by maintaining just enough dispersion of receptive field centers to allow for slightly different preferred tuning of responses to visual space (Lehky and Sereno, 2011). The presence of organized representations of visual space in higher-order regions can still allow for the stimulus size and position invariances frequently described across high-order object- and face-responsive visual regions, as such invariance can arise in regions simply with very large receptive fields (DiCarlo and Maunsell, 2003; Dumoulin and Wandell, 2008; Brewer and Barton, 2012; Haak et al., 2012; Barton and Brewer, 2017). Current research is demonstrating that the majority of higher-order visual areas are organized according to visual space, maintaining retinotopically organized, dispersed RF centers despite increasingly large RF sizes (Hagler and Sereno, 2006; Hagler et al., 2007; Kastner et al., 2007; Swisher et al., 2007; Konen and Kastner, 2008; Arcaro et al., 2009; Lehky and Sereno, 2011; Brewer and Barton, 2012; Lehky et al., 2015; Barton and Brewer, 2017).

Whether the spatial organization remains truly retinotopic or changes to a broader spatiotopic organization—one based on external space rather than retinal space—is still under investigation and cannot be determined with typical visual-field-mapping methods (Sereno et al., 2001; Sereno and Huang, 2006; Hagler et al., 2007; Kastner et al., 2007). In either case, such widespread preservation of visuospatial organization allows for a common reference frame through which information can be passed up or down the visual hierarchy. Theories of attention in which higher-order visual-attentional areas are able to affect many lower-level visual areas simultaneously in spatially specific patterns can be explained through the use of such visual-location-based “channels” (e.g., Sereno et al., 2001; Silver et al., 2005; Saygin and Sereno, 2008; Lauritzen et al., 2009; Silver and Kastner, 2009; Szczepanski et al., 2010). It is also possible that visuospatial organization is maintained despite visual-location information not being critical to the computations of that specific area simply because it would be too disruptive or costly during development to change the organization once it has been established at the level of the retina and earlier visual cortex.

**Figure 10:**
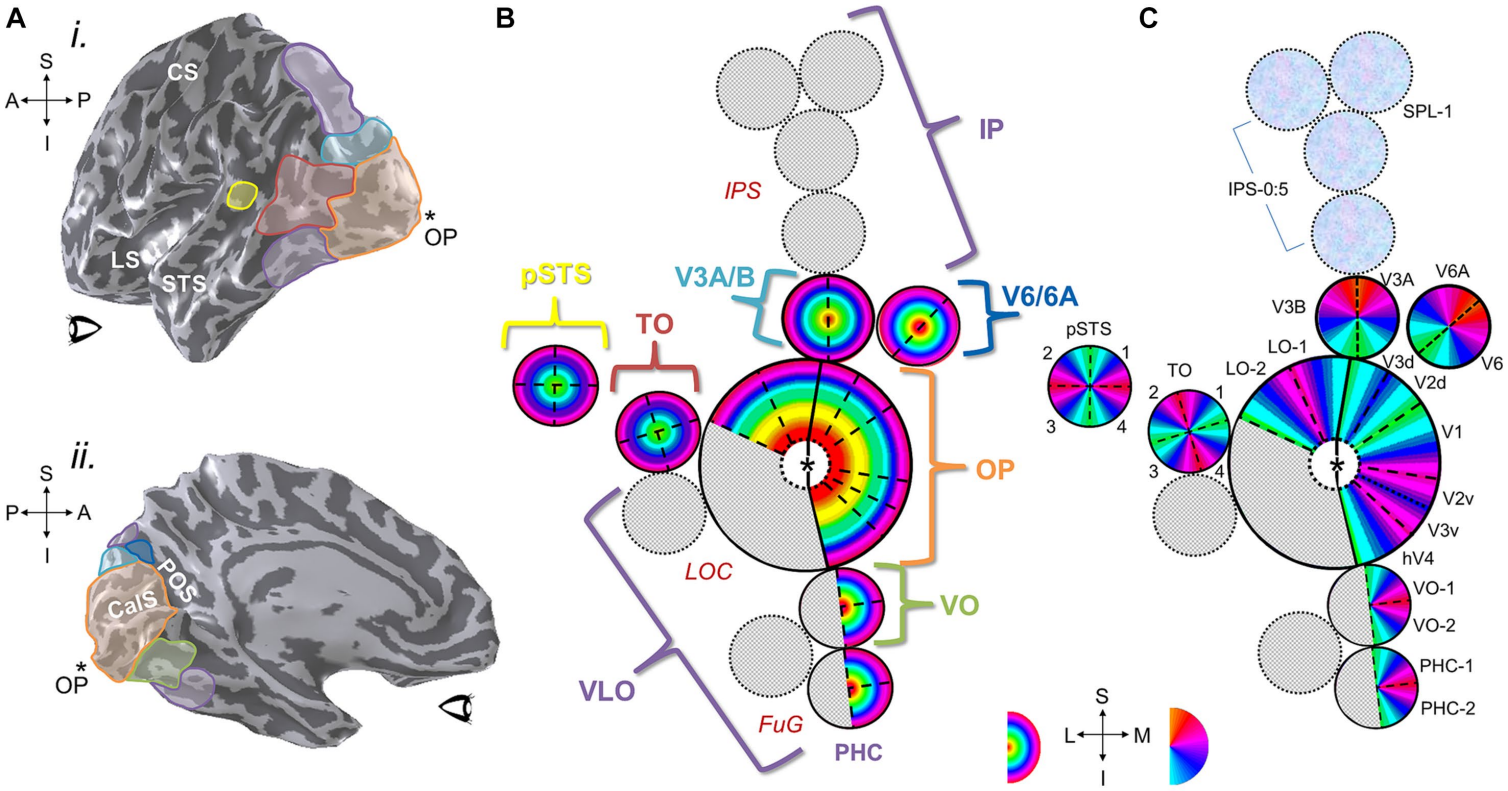
Visual field maps have been defined across much of human visual cortex. **(A) (i)** A left hemisphere from an individual subject is shown as a 3-D inflated rendering in which light gray indicates gyri and dark gray indicates sulci. The positions of several VFM cloverleaf clusters measured in this individual subject are shown along the lateral surface as colored ROIs: orange, OP cluster (occipital pole cluster, lateral subdivision including LO-1, LO-2, LOC; Wandell et al., 2005; Larsson and Heeger, 2006; Brewer and Barton, 2012); red, TO cluster (temporal occipital cluster; also known as hMT+ cluster, human medial temporal complex; Wandell et al., 2005, 2007; Amano et al., 2009; Kolster et al., 2009, 2010; Barton et al., 2012); yellow, pSTS cluster (posterior superior temporal sulcus cluster; Barton and Brewer, 2017); cyan, V3A/B cluster (visual areas 3A and 3B cluster; Press et al., 2001; Wandell et al., 2005; Barton and Brewer, 2017); purple, regions along the dorsal cortex (intraparietal sulcus Schluppeck et al., 2005; Swisher et al., 2007; Silver and Kastner, 2009; Szczepanski et al., 2010) and ventral cortex (fusiform and parahippocampal gyri; Arcaro et al., 2009; Kolster et al., 2010) that are currently under investigation (for reviews, see Wandell et al., 2007; Barton et al., 2012). CS, central sulcus; LS, lateral sulcus; STS, superior temporal sulcus; *OP, occipital pole. Anatomical-directions legend: S, superior; I, inferior; A, anterior; P, posterior. **(ii)** ROIs along the medial surface of the same 3-D-rendered left hemisphere are displayed here, with clusters that span medial and lateral cortex matched in color: orange, OP cluster (medial subdivision including V1, V2, V3, hV4; Wandell et al., 2005; Brewer and Barton, 2012); green, VO cluster (ventral occipital; Brewer et al., 2005; Wandell et al., 2005); cyan, V3A/B cluster; dark blue, V6/6A cluster (Pitzalis et al., 2015); purple, dorsal and ventral regions currently under investigation. POS: parietal-occipital sulcus; CalS: calcarine sulcus. Other details are as in **(i)**. **(B)** Diagram displays eccentricity representations within VFM clusters viewed along a flattened left hemisphere. Color overlays represent the position in visual space that produces the strongest response at that cortical location. Published clusters are labeled in colors corresponding to ROI colors in **(A)**. Regions with cloverleaf clusters currently under investigation or only partially defined are shown in speckled gray with purple labels: IP, intraparietal; VLO, ventral lateral occipital; PHC, parahippocampal cortex. Red anatomical labels: LOC, lateral occipital cortex; FuG, fusiform gyrus; IPS, intraparietal sulcus. Central “*” marks the occipital pole. **(C)** Diagram of polar angle representations viewed on the same schematic of the flattened left hemisphere. Individual VFMs are labeled in black. Blue-magenta textured circles along IPS indicate cortical regions where polar angle representations have been measured, but reliably consistent eccentricity gradients have not yet been published. Other details are as in **(B)**. Bottom inset shows eccentricity color legend *(left)*; approximate anatomical directions for the schematics in **(B,C)** (*middle*); and polar-angle color legend (*right*). Anatomical-directions legend: S, superior; I, inferior; L, lateral; M, medial.

Human visual cortex includes the entire occipital lobe and extends significantly into the parietal and temporal lobes (Figure 10), composing about 20% of cortex (Wandell et al., 2007). The medial wall of occipital cortex in each hemifield contains four hemifield representations of visual space known as V1, V2, V3, and hV4 (for detailed reviews, see Wandell et al., 2007; Brewer and Barton, 2012). V1 consistently occupies the calcarine sulcus, bounded on either side by the split-hemifield representations of V2 and V3 on the lingual gyrus and cuneus. Human V4 (designated hV4 because of the unclear homology to macaque V4) is positioned as a complete hemifield on the ventral occipital surface adjacent to ventral V3 along the posterior fusiform gyrus (Langner et al., 2002; Brewer et al., 2005). These four VFMs compose the medial aspect of the occipital pole cluster (OP cluster), which supports low-level visual computations (Brewer et al., 2005; Wandell et al., 2005; Brewer and Barton, 2012).

Because it receives direct inputs from the retino-geniculate pathway, V1 is considered to be primary visual cortex and is an important site of basic calculations such as orientation, color, and motion (Shapley et al., 2007). Each computation is performed across the entire visual field, yet V1 appears at the level of fMRI measurements to consist of a single, contiguous representation of visual space (Engel et al., 1997; Brewer and Barton, 2012). In essence, V1 is composed of several maps overlaid on one another, each of which performs a single computation (i.e., separated maps for ocular dominance, orientation, and motion; Livingstone and Hubel, 1984; Mikami et al., 1986; Newsome et al., 1986; Movshon and Newsome, 1996; Horton et al., 1997; Koulakov and Chklovskii, 2001). In this arrangement, a very intricate mosaic of neurons subserving these computations allows for each computation to be performed over each portion of the visual field. These mosaics, including pinwheel orientation columns, blobs/interblobs, and ocular dominance columns, have a long history of investigations that are still ongoing (e.g., Livingstone and Hubel, 1984; Bartfeld and Grinvald, 1992; Ohki et al., 2006; Adams et al., 2007; Gilaie-Dotan et al., 2013). These computations divide up into more specialized processing of the visual image after V1, with V2 and hV4 supporting low-level color and form processing, respectively, and V3 playing a role in low-level motion computations (Merigan and Maunsell, 1993; Smith et al., 1998; Wade et al., 2002; Brewer et al., 2005; Larsson et al., 2006; Wandell et al., 2007).

V1, V2, V3, and hV4 each contain a foveal representation positioned at the occipital pole, with progressively more peripheral representations extending into more anteromedial cortex, forming complete eccentricity gradients (Figure 10B; e.g., Sereno et al., 1995; DeYoe et al., 1996; Engel et al., 1997; Wandell et al., 2007). The region where the individual foveal representations meet at the occipital pole is commonly referred to as the foveal confluence (Schira et al., 2009). Despite the apparent merging of these foveal representations into one confluent fovea at the scale of fMRI measurements of eccentricity gradients, distinct boundaries between V1, V2, V3, and hV4 have been shown to be present even within this most central foveal representation (Brewer et al., 2005; Schira et al., 2009, 2010).

The boundaries between each map are delineated by reversals in the polar angle gradients along the medial surface (Figure 10C; e.g., Sereno et al., 1995; DeYoe et al., 1996; Engel et al., 1997; Wandell et al., 2007). V1 has a contiguous polar angle gradient representing a full hemifield, while V2 and V3 have split-hemifield representations (i.e., quarterfields), which are named by their positions ventral or dorsal to V1: V2d, V2v, V3d, V3v. Because of their relatively consistent anatomical locations and unique concentric polar angle gradients, these three VFMs are typically the first landmarks identified in visual-field-mapping analyses (Engel et al., 1994; Sereno et al., 1995). However, as noted above, the surface areas of these three VFMs fluctuate significantly among individuals independent of overall brain size (Dougherty et al., 2003). While V1 is always located along the fundus and up the walls of the calcarine sulcus in normal individuals, an increase in V1 size will consequently shift the specific positions of V2 and V3 along the neighboring gyri and sulci. VFMs beyond V3, such as the contiguous hV4 hemifield, continue to shift variably along the cortical surface in accordance with variable individual VFM sizes (Brewer et al., 2005; Winawer et al., 2010).

This pattern of VFMs continues across most if not all visual cortex, with loose divisions of processing into dorsal and ventral streams for the perception of action and recognition, respectively (Figure 10; Mishkin and Ungerleider, 1982; Goodale and Milner, 1992; Van Essen, 2003; Lehky and Sereno, 2007; Gilaie-Dotan et al., 2013). Groups of VFMs are then organized into cloverleaf clusters that are now either completely or partially defined (Wandell et al., 2005; Kolster et al., 2010; Brewer and Barton, 2012). Within each cluster, eccentricity representations run from foveal representations at the center of the cluster to peripheral representations at the outskirts of the cluster. Thus, boundaries between clusters are defined as reversals in eccentricity representations (Figure 10B). Boundaries between VFMs within a cluster occur in reversals of polar angle representations, typically along a representation of the vertical meridian of visual space except for the split quarterfield dorsal/ventral maps of V2 and V3, which are divided along the horizontal meridian (Figure 10C).

Along these dorsal and ventral streams, the medial-occipital VFMs of V1, V2, V3, and hV4 combine with the lateral VFMs LO-1 and LO-2 and a small number of yet-undetermined VFMs to form the occipital pole (OP) cloverleaf cluster, centered on its namesake (Wandell et al., 2005; Larsson and Heeger, 2006; Larsson et al., 2006; Montaser-Kouhsari et al., 2007; Kolster et al., 2010; Brewer and Barton, 2012). While the medial maps are well-established areas involved in the early stages of visual processing, the lateral VFMs in this cluster are likely involved with various stages of processing for visual object recognition and are still under extensive study. Superior to the OP cluster is first the two-map V3A/B cluster along the transverse occipital sulcus that plays a role in mid-level motion processing (Tootell et al., 1997; Press et al., 2001; Barton and Brewer, 2017). Along the medial wall in this region anterior to V3A/B and V3d is the two-map putative cluster of V6 and V6A (Pitzalis et al., 2006, 2015). The V6 and V6A VFMs are thought to be involved in evaluating object distance during self-motion and planning pointing or reaching responses in response, respectively (Fattori et al., 2009; Pitzalis et al., 2010, 2013). Further superior/anterior to these regions along the inferior parietal sulcus (IPS) are several putative clusters that include VFMs currently called IPS-0 (or V7) to IPS-5 and SPL-1 (Sereno et al., 2001; Schluppeck et al., 2005; Silver et al., 2005; Kastner et al., 2007; Konen and Kastner, 2008; Lauritzen et al., 2009; Silver and Kastner, 2009; Brewer and Barton, 2018). These parietal VFMs overlie regions involved in attention and working memory, as well as various aspects of sensorimotor integration. Likely due to their roles in these cognitive processes, these IPS regions beyond V3A/B are increasingly affected by changes in attention, with VFMs often unable to be measured without the proper attentional controls included in the visual stimuli (Silver et al., 2005; Saygin and Sereno, 2008). In addition, the majority of the IPS maps do not yet have published eccentricity representations, so the final organizations of each cluster remains to be determined (Brewer and Barton, 2012).

Anterior to the OP cluster along the lateral surface is the four-map temporal occipital (TO) cluster, alternatively called the hMT+ complex or cluster, a key cortical region for visual motion processing (Huk et al., 2002; Amano et al., 2009; Kolster et al., 2010). Further anterior is the recently discovered four-VFM posterior superior temporal sulcus (pSTS) cluster that is likely involved in multisensory integration (Barton and Brewer, 2017). Inferior to the OP cluster is the ventral occipital (VO) cluster, which currently contains two measured VFMs (VO-1 and VO-2) in a likely set of four (Wade et al., 2002; Brewer et al., 2005) and processes higher-level visual form and color information. Finally, anterior to the VO cluster along the ventral surface is the parahippocampal cortex (PHC) cluster, which also has two currently measured VFMs (PHC-1 and PHC-2) that likely also form a group of four maps (Arcaro et al., 2009). The PHC cluster is thought to play a role in visual scene perception, consistent with role of this ventral stream region established from other measurements as well (Grill-Spector et al., 2017; Epstein and Baker, 2019).

Due to the long history of extensive research into VFMs in human and animal models as well as complications with neuroimaging measurements for the non-visual senses, we have a vastly better understanding of the organization of CFMs in the visual system than the other senses (Brewer and Barton, 2018). As such, the patterns we observe in visual cortex can serve to varying degrees as the foundation for our expectations in the other sensory systems. As we will review next, new research is starting to reveal similar topographical representations, CFMs, cloverleaf clusters, and/or dorsal/ventral streams in human auditory, somatosensory, and gustatory cortex.

### 4.2 Auditory field maps

**Figure 11:**
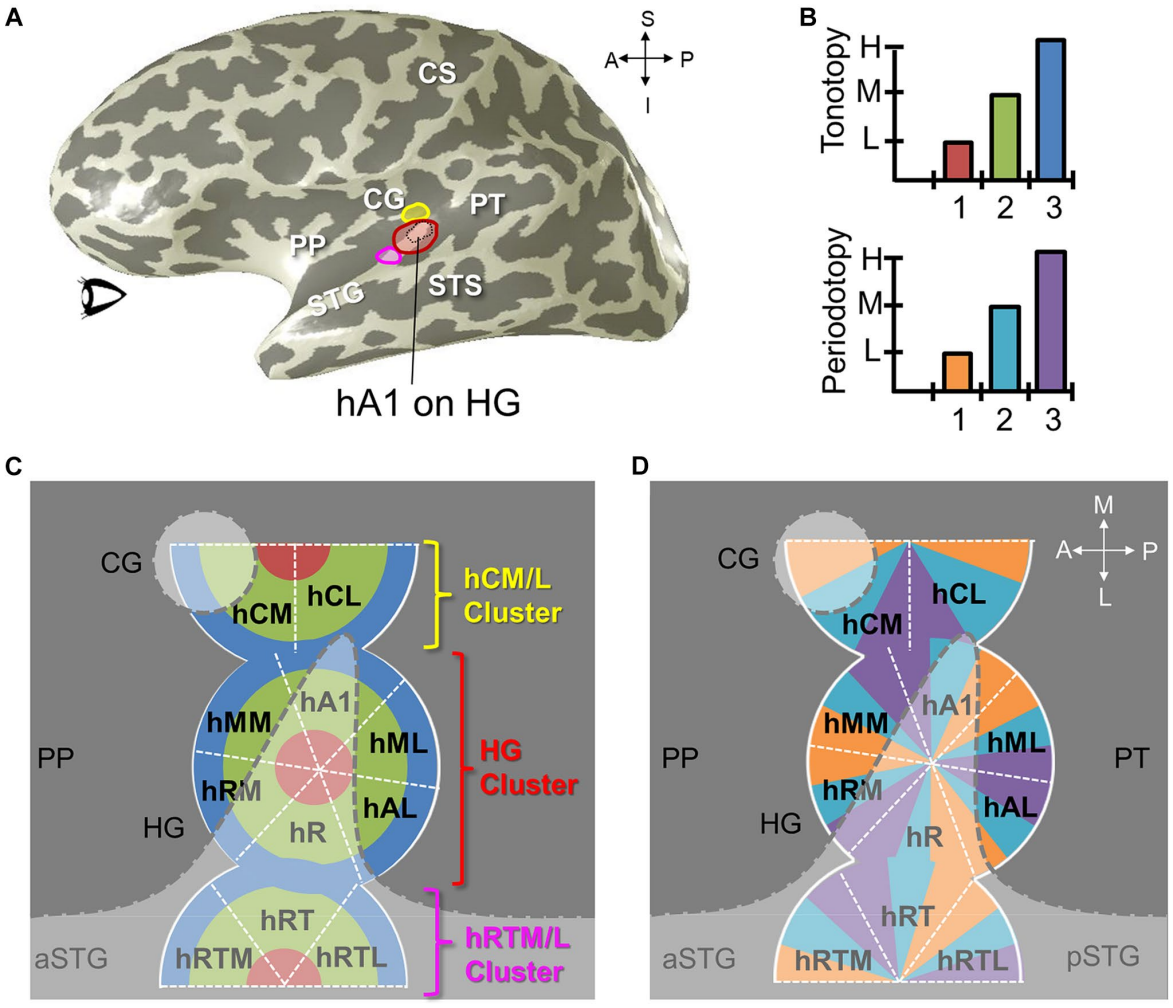
Auditory field maps have been defined in the core and belt regions of human auditory cortex. **(A)** The 3-D inflated rendering of an individual left hemisphere lateral surface is shown with light gray gyri and dark gray sulci. This subject’s hA1 auditory field map (AFM) is labeled with the black dotted lines at the tip of Heschl’s gyrus (HG). The three colored ROIs on HG denote the locations of the cloverleaf clusters comprising the core and belt AFMs: Yellow, hCM/hCL cluster; Red, HG cluster including hA1, hR, hRM, hMM, hML, hAL; Magenta, hRTM/hRT/hRTL cluster (Barton et al., 2012; Brewer and Barton, 2016b). CS, central sulcus; STG, superior temporal gyrus; STS, superior temporal sulcus. CG, circular gyrus; PP, planum polare; PT, planum temporale. Anatomical-directions legend: S, superior; I, inferior; A, anterior; P, posterior. **(B)** Schematics depict the color code for the two orthogonal dimensions that are required to define an auditory field map: tonotopy (*top*), periodotopy (*bottom*). Diagrams in **(C,D)** use these colors for tonotopic and periodotopic representations, respectively. **(C)** A model of tonotopic representations of core and belt auditory field maps is overlaid on a schematic of a flattened region of cortex around HG. Dark gray indicates the plane of the lateral sulcus, while light gray indicates the circular gyrus (CG), Heschl’s gyrus (HG), and the superior temporal gyrus (STS; a/pSTS, anterior/posterior STS). White dotted lines denote the approximate boundaries between individual AFMs. AFM cloverleaf clusters are labeled to match those in **(A)**. **(D)** A model of periodotopic representations of core and belt auditory field maps is overlaid on the same schematic of a flattened region of cortex around HG. Other details are as in **(C)**. Upper right inset displays the approximate anatomical axes: M, medial; L, lateral; A, anterior; P, posterior.

Auditory stimuli are fundamentally spectrotemporal, meaning that complex sound waves have two fundamental components important for human perception: spectral information – such as which frequencies are present in the sound waves, and temporal information – such as when and for how long those frequencies are present (Shamma, 2001). Auditory field maps (AFMs), much like VFMs, are composed of two orthogonal dimensions representing each of these spectral and temporal components of sound (Barton et al., 2012; Herdener et al., 2013; Brewer and Barton, 2016b; Figure 11). It is important to note that these topographical spectral and temporal representations of AFMs are not associated with auditory spatial information; we do not yet know how auditory space—that is, where sounds are occurring around us—is encoded in human cortex after processing in the brainstem (Brewer and Barton, 2018). Thus we currently discuss spatial mapping for the visual and somatosensory systems, but frequency mapping of two types for audition.

For many years, only one spectrotemporal dimension—tonotopy (or “cochleotopy”)—was able to be measured in human cortex (for discussions, see Wessinger et al., 2001; Barton et al., 2012; Ress and Chandrasekaran, 2013; Brewer and Barton, 2016a; Chang et al., 2017). Tonotopy reflects the organization of the cochlea, which transduces complex sound waves into streams of neural signals representing the intensity of each frequency, analogous to a Fourier analysis (Moerel et al., 2012). Higher frequencies are transduced near the entrance of the cochlea, while continually decreasing frequencies are transduced further into the membrane, creating similar topographical gradients of frequency representation in human cortex that repeat with each AFM (Formisano et al., 2003; Humphries et al., 2010). More recently, it has been demonstrated that periodotopy, which represents the temporal information present in complex sound waves, known as periodicity, is the orthogonal counterpart to tonotopy that allows for the correct definition of AFMs according to principles consistent with the well-characterized VFMs of the visual system (Schreiner and Langner, 1988; Langner, 1992; Langner et al., 1997; Schulze et al., 2002; Baumann et al., 2011; Barton et al., 2012; Herdener et al., 2013). More specifically, periodotopic gradients in human cortex consist of neurons that code periodicity information by time-locking to the amplitude modulation of the sound wave (e.g., the length of time from one peak to the next of the temporal envelope; Shamma, 2001). Other neurons activated by periodotopic stimuli likely include neurons selective for the onset and offset of sound waves with varying refractory times as well as neurons that respond to differing sound-wave durations. Regardless of what aspect of periodicity the neurons are specifically encoding, periodotopic gradients are organized topographically along human cortex from lower modulation rates to higher ones. Similar measurements have also been observed in macaque monkey (Baumann et al., 2011).

Investigation into the types of computations performed by the currently known AFMs in human cortex has been limited both by the initial, incorrect use of tonotopic gradients alone to define AFM boundaries as well as little research to date into specific human AFM functions (Brewer and Barton, 2016b). At this time, the nomenclature used to name human AFMs and set up expectations for their functions is based on likely homologs to areas defined in non-human primate models (Barton et al., 2012). The presumed anatomical homologs between macaque and human and similar organization of tonotopic gradients provide converging evidence for the definition of matching AFMs in human cortex. Measurements in macaque define an auditory “core” consisting of three primary auditory areas: A1 (primary auditory cortex), R (rostral area), and RT (rostrotemporal area), differentiated from surrounding cortex based largely on the density of inputs from the thalamus to each area in the core as well as their more basic response characteristics (Merzenich and Brugge, 1973; Pandya and Sanides, 1973; Galaburda and Sanides, 1980; Galaburda and Pandya, 1983; Sweet et al., 2005; Dick et al., 2012; Brewer and Barton, 2016a). Lateral (CL, CM, AL, RTL) and medial (CM, RM, MM, RTM) “belt” regions surrounding the core are thought to be the next stages of auditory processing (Rauschecker et al., 1995; Rauschecker and Tian, 2004; Tian and Rauschecker, 2004; Kusmierek and Rauschecker, 2009). Finally, tertiary orders of the hierarchy consist of at least two further lateral “parabelt” regions, which have broad connections among various other auditory and multimodal regions of cortex (Kaas and Hackett, 2000; Kajikawa et al., 2015). While these macaque auditory regions lie along macaque superior temporal gyrus (STG), the human homologs are centered on Heschl’s gyrus (HG), which is rotated medially relative to human STG (Figure 11A; Leonard et al., 1998; Morosan et al., 2001; Barton et al., 2012; Dick et al., 2012). Thus the human names lack the directional implications of the original macaque ones. For example, while R is rostral to A1 in macaque and is thusly named, human R is lateral to human A1 due to the rotation of likely human homologs along HG relative to macaque STG (Barton et al., 2012; Brewer and Barton, 2016b). To account for these differences and the possibility that AFM functions differ between the species, the human auditory areas have been named with “h” before the macaque designation. Thus hA1 is the likely human homolog to macaque A1 and was identified in human as such through a combination of its location, smaller RF sizes, and internal cortical magnification that represents a wide span of frequencies.

The additional information contained in the periodotopic gradients, when combined with the tonotopic gradients to form complete AFMs, indicates that the core vs. belt macaque organizational model, while useful, is insufficient to fully describe the data observed in human cortex. Instead, AFMs appear to be organized into at least three cloverleaf clusters, similar to the organization found in VFMs of the visual system (Brewer and Barton, 2018; Figures 11C,D). Of these, one complete cluster has been measured, the HG cluster, which consists of hA1, hML, hAL, hR, hRM, and hMM, while hCM and hCL likely form part of another cluster and hRT, hRTL, and hRTM form part of a third likely cluster. Reversals in the periodotopic gradients divide the clusters into individual VFMs, while reversals in the tonotopic gradients divide each cluster from its neighbors. The discovery that AFMs are organized into cloverleaf clusters like VFMs indicates that cloverleaf clusters are a fundamental organizing principle of sensory cortex, likely to exist across sensory modalities (Brewer and Barton, 2018).

### 4.3 Somatotopic representations

**Figure 12:**
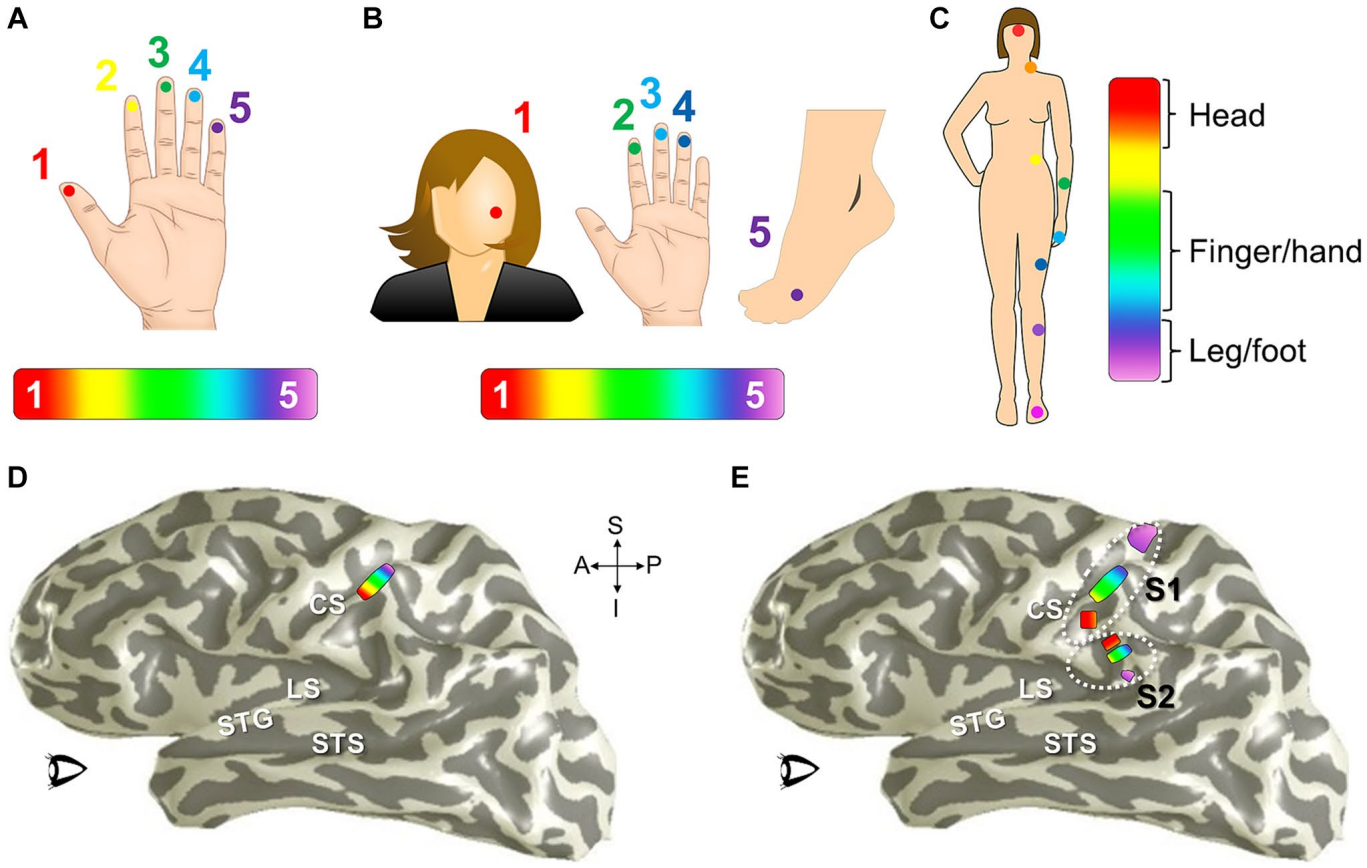
One dimension of human somatosensory field maps has been explored for tactile and pain representations for selected body parts in S1 and S2. **(A)** Hand diagram denotes the locations and order of fingertip stimulation by fine-touch (e.g., air puff or vibration) or nociceptive (e.g., radiant-heat laser) stimuli typically used by traveling-wave paradigms (e.g., Sanchez-Panchuelo et al., 2010; Mancini et al., 2012; Kolasinski et al., 2016a). Color legend is shown for stimulation of fingertips 1 to 5. **(B)** Schematic displays where measurement points have been tested for larger range somatotopy on face, fingertips, and foot in Sanchez Panchuelo et al. (2018). Note that the color scheme now represents different body locations than in **(A)**. **(C)** Body diagram shows points of somatotopic measurement from head to foot from Willoughby et al. (2020). The color-grouping for head, finger/hand, and leg/foot representations loosely matches the color scheme from **(B)**. **(D)** A color-coded schematic of cortical responses to the fingertip stimuli from **(A)** is overlaid on a 3-D inflated left hemisphere to display the approximate location of fingertip somatotopy in S1. These fingertip representations are estimated from individual-subject and group-averaged responses to both the light touch and pain stimuli from Sanchez-Panchuelo et al. (2010) and Mancini et al. (2012). **(E)** A color-coded schematic of cortical responses to the head-hand-foot somatotopic measurements from **(B,C)** is overlaid on the same 3-D inflated left hemisphere to display the approximate location of these somatotopic representations in both S1 and likely S2. These coarse somatotopic representations were estimated from individual-subject data shown in Sanchez Panchuelo et al. (2018) and Willoughby et al. (2020). White dotted lines approximate the locations of human primary somatosensory cortex (S1) and secondary somatosensory cortex (S2). CS, central sulcus; LS, lateral sulcus; STG, superior temporal gyrus; STS, superior temporal sulcus. Light gray, gyri; dark gray, sulci. Anatomical-directions legend: S, superior; I, inferior; A, anterior; P, posterior.

Somatosensation is an overarching term for several subtypes of sensation, which include mechanoreception (e.g., vibration, discriminatory/fine touch, deep pressure), nociception (e.g., pain); thermoception (e.g., temperature); equilibrioception (e.g., balance); and proprioception (e.g., body position/movement; Lumpkin and Bautista, 2005; Eickhoff et al., 2006; Kaas, 2012; Pleger and Villringer, 2013). Somatosensory processing begins with peripheral receptors in the skin, organs, joints, and tissues that often have evolved highly specialized structures for optimizing their ability to detect changes in the environment, with receptor locations along the skin producing a somatotopic map of the body surface. These specialized responses then follow associated parallel processing pathways of somatosensory information through the spinal cord, to the brain stem, several nuclei of the thalamus, and ultimately to various cortical and other subcortical regions (Penfield and Boldrey, 1937; Krubitzer et al., 1995a; Kaas, 1997; Kaas, 2012; Saadon-Grosman et al., 2020a; Willoughby et al., 2020). In human and related animal models, these regions include the primary somatosensory cortex (S1 or “SI”) along the posterior bank of the central sulcus and the postcentral gyrus (Figure 12) and the secondary somatosensory area (S2 or “SII”) abutting the inferior part of S1 on the superior bank of the lateral fissure. Additional somatosensory representations have been measured in the superior and inferior parietal lobules, cingulate cortex, inferior frontal gyrus, and the frontal operculum (Ruben et al., 2001; Hagen et al., 2002; Young et al., 2004; Arienzo et al., 2006; Huang et al., 2012).

Investigations of somatotopic representations in human cortex to date have focused primarily on a few types of mechanosensation and nociception (Sanchez-Panchuelo et al., 2010; Mancini et al., 2012; Sanchez Panchuelo et al., 2018; Willoughby et al., 2020). Similar to the retinal information in the visual system, somatosensory information about tactile and pain stimuli from the surface of the skin arises from a relatively easy-to-conceptualize, 2D space along the body (Rothschild and Mizrahi, 2015). In contrast to the visual system, however, systematically stimulating significant regions of this skin space to map out the associated cortical sensory topography in humans is much more difficult experimentally (Disbrow et al., 2000; Ruben et al., 2001; Sanchez-Panchuelo et al., 2010; Mancini et al., 2012; Besle et al., 2013; Willoughby et al., 2020). For example, devices that produce high-resolution, light-touch stimulation of large skin regions are difficult to create and even more difficult to adapt to an MR-scanning environment. In addition, the cortical anatomy along the central sulcus presented significant problems for accurate neuroimaging measurements for many years. S1 lies along the postcentral gyrus just millimeters from a similar somatotopic map in primary motor cortex (M1) on the closely abutting precentral gyrus (Disbrow et al., 2000; Ruben et al., 2001; Eickhoff et al., 2007). With the juxtaposition of these two similar topographies across the central sulcus, typical traveling-wave CFM measurements at lower magnetic-field strengths have generally been insufficient to precisely resolve the somatotopic organization along each gyrus (Besle et al., 2013). The spatial resolution of most of these fMRI measurements was not high enough until recently to overcome the partial-volume effects of a single voxel combining measurements of neuronal responses from S1 and M1 into one data point (Gonzalez Ballester et al., 2002; Sanchez-Panchuelo et al., 2010; Willoughby et al., 2020). In addition, somatosensory maps in both adult human and other mammals appear to be part of a rather dynamic system that can undergo significant alterations across much of the lifespan, in contrast to the visual system, in which the cortical plasticity of VFMs is greatly reduced after the close of the critical period of visual development (e.g., Kaas et al., 1983; Kaas, 1991; Smirnakis et al., 2005; Jain et al., 2008; Wandell and Smirnakis, 2009; Brewer and Barton, 2014; Qi et al., 2014; Barton and Brewer, 2015; Kolasinski et al., 2016b). Finally, relatively high variability of the topographies in this area across human subjects caused significant issues for the many fMRI studies that employed group-averaging to the measurements, which compounded the partial volume effects with additional blurring of the data from the averaging (Duncan and Boynton, 2007; Sanchez-Panchuelo et al., 2010; Brewer and Barton, 2012; Besle et al., 2014; Sánchez-Panchuelo et al., 2014; Kolasinski et al., 2016a).

Because of these issues, a significant amount of our knowledge from the last century regarding the localization of functions in the human somatosensory cortex has relied on lesion studies and intra-operative neuronal recording and stimulation measurements in human patients together with examinations of S1, S2, and related areas in various animal models (e.g., Penfield and Boldrey, 1937; Woolsey et al., 1979; Krubitzer et al., 1995a,b; Roux et al., 2018; Saadon-Grosman et al., 2020b). Penfield and colleagues’ intraoperative experiments on humans in the 1930s have served since then as the foundation for our current expectations of S1 and S2 organization in human, despite early concerns about reproducibility those same researchers raised regarding the concept of their cortical homunculus (Penfield and Boldrey, 1937; Snyder and Whitaker, 2013; Saadon-Grosman et al., 2020b; Willoughby et al., 2020). Researchers have also questioned what differences may arise for cortical responses activated by such direct stimulation of S1 with electrodes in cortex, which bypasses the peripheral nerves, in contrast to those from the normal physiological stimulation of S1 through the peripheral receptors in the skin.

Measurements subsequent to the Penfield studies have generally supported the idea of the somatotopic homunculus running medial-laterally—one topographical dimension—in human cortex, but it was just recently that research began to make progress at measuring the details of the two-dimensional topography and cortical magnification of specific body-part representations (e.g., face and hands) within S1 and S2 in higher resolution (Penfield and Boldrey, 1937; Roux et al., 2018; Schellekens et al., 2018; Willoughby et al., 2020). In the 1990s and early 2000s, several studies using both fMRI and neuromagnetic methodologies began to map out the two-dimensional representations of skin regions along the palm and/or fingertips. Despite the relatively lower spatial resolution measurements from the available technology at the time, these researchers were able to demonstrate rostal-caudal (or proximal-distal) topographical gradients for light touch, vibrations, and innocuous electrical stimulation in addition to the medial-lateral gradients of the homunculus in human S1 (Hari et al., 1993; Gelnar et al., 1998; Hashimoto et al., 1999; Francis et al., 2000; Kurth et al., 2000; Deuchert et al., 2002; Blankenburg et al., 2003). Importantly, Blankenburg et al. (2003) defined rostal-caudal gradients along the finger and palm representation that included a mirror reversal of the somatotopic gradients at the fingertip representations from Brodmann’s area 3b to area 1, two cytoarchitectural subdivisions of S1 that display preferential responses to the stimulation of cutaneous receptors (Powell and Mountcastle, 1959; Iwamura et al., 1993). As in the visual system, such gradient reversals should reflect multiple representations of the skin topography, perhaps with each map dedicated to different tactile modalities or levels of complexity of somatosensory processing (Iwamura et al., 1993; Friedman et al., 2004). Exactly how these representations form complete somatosensory field maps (SFMs) integrating the multiple tactile and nociceptive modalities remains to be seen, with larger scale mapping of the skin surface likely necessary to resolve these questions.

The recent high-magnetic-field and high-resolution (at 3 T and 7 T) fMRI experiments measuring S1 and S2 have made excellent progress at beginning to map out detailed regions of somatotopic organization in human non-invasively and using peripheral—and thus potentially more natural—somatosensory stimulation (e.g., vibrotactile, pneumatic, nociceptive; Sanchez-Panchuelo et al., 2010; Mancini et al., 2012; Sánchez-Panchuelo et al., 2014; Sanchez Panchuelo et al., 2018; Saadon-Grosman et al., 2020b; Willoughby et al., 2020). Converging evidence from several independent labs has demonstrated a pattern of responses for leg/foot, finger/hand, and head representations for each area that mostly matches our expectations from the prior work (Figure 12B). The most detailed examinations have been of the fingertips, with evidence for overlapping topographies for vibrotactile, pneumatic, and pain stimulation in S1 (Sanchez-Panchuelo et al., 2010; Mancini et al., 2012). Interestingly, there are suggestions that the topographies for pain and tactile inputs differ somewhat in their cortical magnification despite the overlapping location (Mancini et al., 2012). In addition, the size of the topographies of the fingertips has been correlated with tactile acuity (Duncan and Boynton, 2007). There is some suggestion that differences may exist between these maps and those initially proposed by Penfield, but the general pattern of the homunculus is largely consistent, and it is difficult to fully compare the two measurement types with the necessarily limited body-coverage of stimulation in the current studies (Saadon-Grosman et al., 2020b; Willoughby et al., 2020). Much as we see reversals from one VFM or AFM to the next, the regions of S1 abutting S2 appears to occur in these measurements at a representation of the face/head. Finally, evidence is emerging that the somatosensory system is also loosely organized around the same dorsal/ventral perceptual streams as we see in vision and audition/language (Goodale and Milner, 1992; Hickok and Poeppel, 2007; Saadon-Grosman et al., 2020a).

It is important to remember that, in contrast to visual and auditory CFMs, somatosensory representations in human cortex have only been measured with fMRI to date in these experiments as a few discrete points across the body (Figure 12A). The discrete measurement points produce a one-dimensional gradient running head to foot and/or across the tips of the fingers, rather than a map of the 2D space of the entire skin, so these regions cannot yet be termed somatosensory field maps (SFMs). A full SFM will require larger-scale mapping of two body axes, which could be defined as any paired combination of superior–inferior (rostral-caudal), anterior–posterior, medial-lateral, or distal-proximal gradients. The use of discrete points also necessarily means that determinations of differences in cortical magnification across the somatotopic map cannot yet be precisely measured (Engel et al., 1997; Brewer and Barton, 2016b). With discrete, non-abutting measurement points in a traveling-wave paradigm, the spread of the cortical response to neighboring regions that are slightly—but not preferentially—activated by the stimulation point produces an enlarged map of that region (Engel et al., 1997; Brewer et al., 2005; Wandell et al., 2007; Brewer and Barton, 2012). As our measurements advance, it will be exciting to delineate exactly how cortical magnification differs among body regions and across individuals as well as whether the putative SFMs are also arranged into a macrostructural pattern of cloverleaf clusters.

### 4.4 The chemical senses: topographies in gustation and olfaction

**Figure 13:**
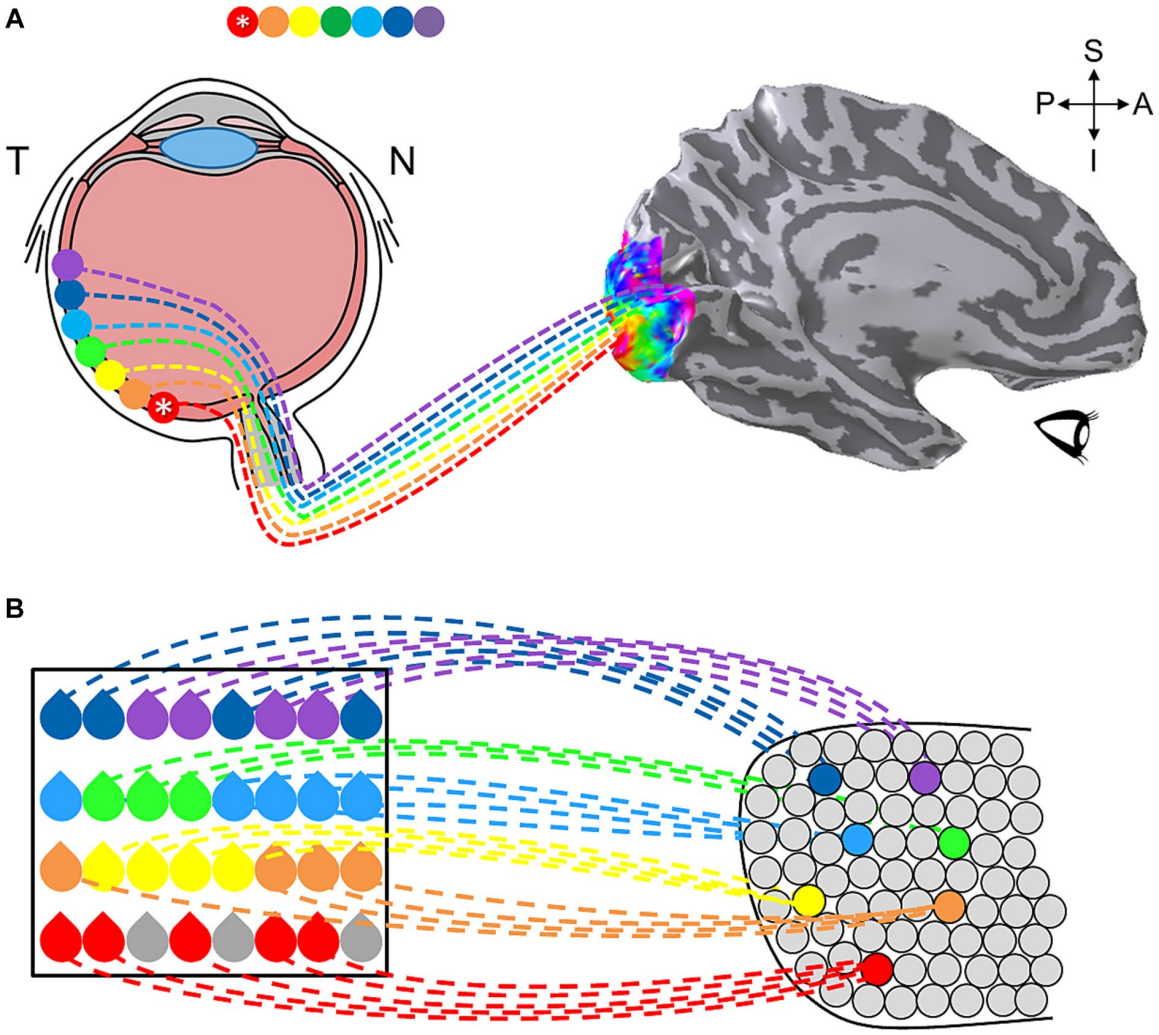
Olfactory topography may compose a discrete map vs. a continuous one. **(A)** The schematic shows an example of the continuous retinotopic map of the visual system. The diagram of the left eye is fixated on the asterisk in the red circle of the rainbow in the right visual hemifield. The light from the rainbow travels across the eye to be absorbed by the photoreceptors of the opposite, temporal side of the retina (T, temporal; N, nasal). The asterisk on the retina represents the central fovea. Neighboring points in the visual field thus activate neighboring points on the retina of the stabilized eye. The retinal ganglion cells at each retinal location maintain this retinotopic organization through their axons that project to the thalamus and then, after synapsing, to V1. The rainbow eccentricity pattern is shown from visual space, to the retina, through the axons of the optic nerve and tract, and to the colored data overlay that demonstrates the continuous eccentricity map along the medial wall of the inflated left hemisphere. Other details are as in Figure 10. **(B)** This diagram displays a schematic of a discrete olfactory map. The black square (*left*) represents a region of olfactory epithelium, with each color drop denoting an olfactory receptor neuron expressing a specific receptor type. Rather than projecting to a continuous map of stimulus dimensions, the olfactory sensory neurons expressing the same odorant receptor converge to form glomeruli in specific locations of the olfactory bulb (*right*). Thus the discrete topographic organization in this sensory system is based on the type rather than the spatial distribution of the sensory inputs. Rainbow colors represent a subset of specific odorant receptor types, while gray circles represent additional odorant receptor types not depicted with connections in this diagram.

Only recently has research begun to unravel the cortical representations of the chemical senses, gustation (taste) and olfaction (smell). In the case of vision, audition, and somatosensation, there is a general understanding of which values of the sensory stimulus should be represented in a contiguous topographical cortical representation. In contrast, the mapping of the chemical senses presents a more challenging task, as the stimuli are composed of molecules that exhibit a wide range of diversity across such factors as size, charge distribution, bond saturation, functional groups, and three-dimensional structure (e.g., Chen et al., 2011; Lundstrom et al., 2011; Murthy, 2011; Auffarth, 2013; Miskovic and Anderson, 2018). Because a single, small molecule can be characterized by numerous parameters, it is nearly impossible to systematically map even a fraction of these parameters onto a 2D surface without some knowledge of what privileged parameters might have been selectively favored during evolution to serve as a fundamental organizing principle. In addition, such chemotopic organization may be more likely to produce a discrete map that combines similar receptor or molecular inputs onto target neurons rather than the continuous topographical gradients we expect for the other CFMs (Figure 13; Luo and Flanagan, 2007; Murthy, 2011; Francia and Lodovichi, 2021).

While the quality of such chemical stimuli does not inherently provide a topographical criterion to predict which region of the cortex would preferentially represent a specific tastant or olfactant, it is still possible that a spatially segregated and ordered cortical representation of these molecules’ qualities may arise from genetically predetermined neural circuits in specific regions, such as those contributing to innate ecological behaviors (Luo and Flanagan, 2007; Sosulski et al., 2011; Chikazoe et al., 2019; Olofsson and Freiherr, 2019). For example, taste receptors are finely tuned to recognize specific taste types associated with distinct hedonic values and thus play a vital role in guiding food selection through reward and punishment (Rolls, 2011; Berridge and Kringelbach, 2015). Sweet receptors facilitate the identification of energy-rich nutrients like glucose, while bitter receptors are thought to serve as protection against potentially harmful substances, forming the basis of oral aversion and disgust (Accolla et al., 2007; Peng et al., 2015). Because the ability to identify food as safe to eat by taste or smell is crucial for the survival of living organisms, aspects of these senses tend to be highly conserved across species. Furthermore, their cortical processing may fine-tune each taste or smell domain to identify particular nutrients, toxic substances, chemicals associated with physiological functions, and/or hedonic attributes (Lundstrom et al., 2011; Miskovic and Anderson, 2018).

#### 4.4.1 Gustatopic representations

The mammalian tongue possesses specialized receptors that are attuned to fundamental taste categories. In humans, five primary tastes are perceived: sweet, sour, umami, bitter, and salt. There are additional potential basic tastes, such as CO_2_, fat, water, pungency (e.g., spiciness or hotness), coolness, calcium, or metallicness (Chaudhari and Roper, 2010; Crouzet et al., 2015). Interestingly, CO_2_ contributes to the taste of carbonation through a dedicated taste-receptor mechanism (Chandrashekar et al., 2009). Such taste perception originates in the tongue from taste buds located on the circumvallate, foliate, and fungiform gustatory papillae (Roper, 2013). Taste information in mammals then follows multiple ascending pathways in the brainstem and ultimately activates primary taste cortex thought to be in the insular cortex, with potential secondary taste areas in the operculum (Sugita and Shiba, 2005; Kaas et al., 2006; Rolls, 2006; Chen et al., 2011; Stevenson et al., 2013). There are two primary conflicting theories of how taste information is encoded and transmitted to cortical gustatory processing (Mueller et al., 2005; Breslin and Huang, 2006; Huang et al., 2006; Chandrashekar et al., 2010). In the labeled-line model, information about a single taste type is encoded by a dedicated set of receptor cells specifically tuned for that taste. This single-taste information is then conveyed to gustatory cortex through taste-specific afferent fibers. In contrast, the across-fiber-pattern model proposes that taste information is communicated across multiple afferent fibers coding taste-type information via population codes of spatiotemporal patterns.

While the primary gustatory cortex in other animals has been shown to distinctly represent these taste categories, identifying them in humans was challenging for many years (Sugita and Shiba, 2005; Accolla et al., 2007; Chen et al., 2011, 2021). Over the last decade, gustatory stimuli in human have been shown to activate various cortical areas, including the insula, frontal operculum, parietal operculum, and orbitofrontal cortex. In addition, other measurements have demonstrated that the human insula represents at least two interrelated gustatory parameters: taste qualities and their palatability (i.e., trial-by-trial hedonic responses; Chikazoe et al., 2014; Crouzet et al., 2015). The insula also receives sensory inputs from visceral organs, including information about gastric distension, temperature, and pain, which may overlap with inputs originating from the chemosensory receptors in the tongue and oral cavity, to generate a comprehensive interoceptive system (Craig, 2009). The integration of these separate gustatory inputs into a comprehensive cortical representation, along with essential inputs from the other sensory modalities, could facilitate our intricate experiences of palatability and higher-order flavor perception (Yeshurun and Sobel, 2010a).

**Figure 14:**
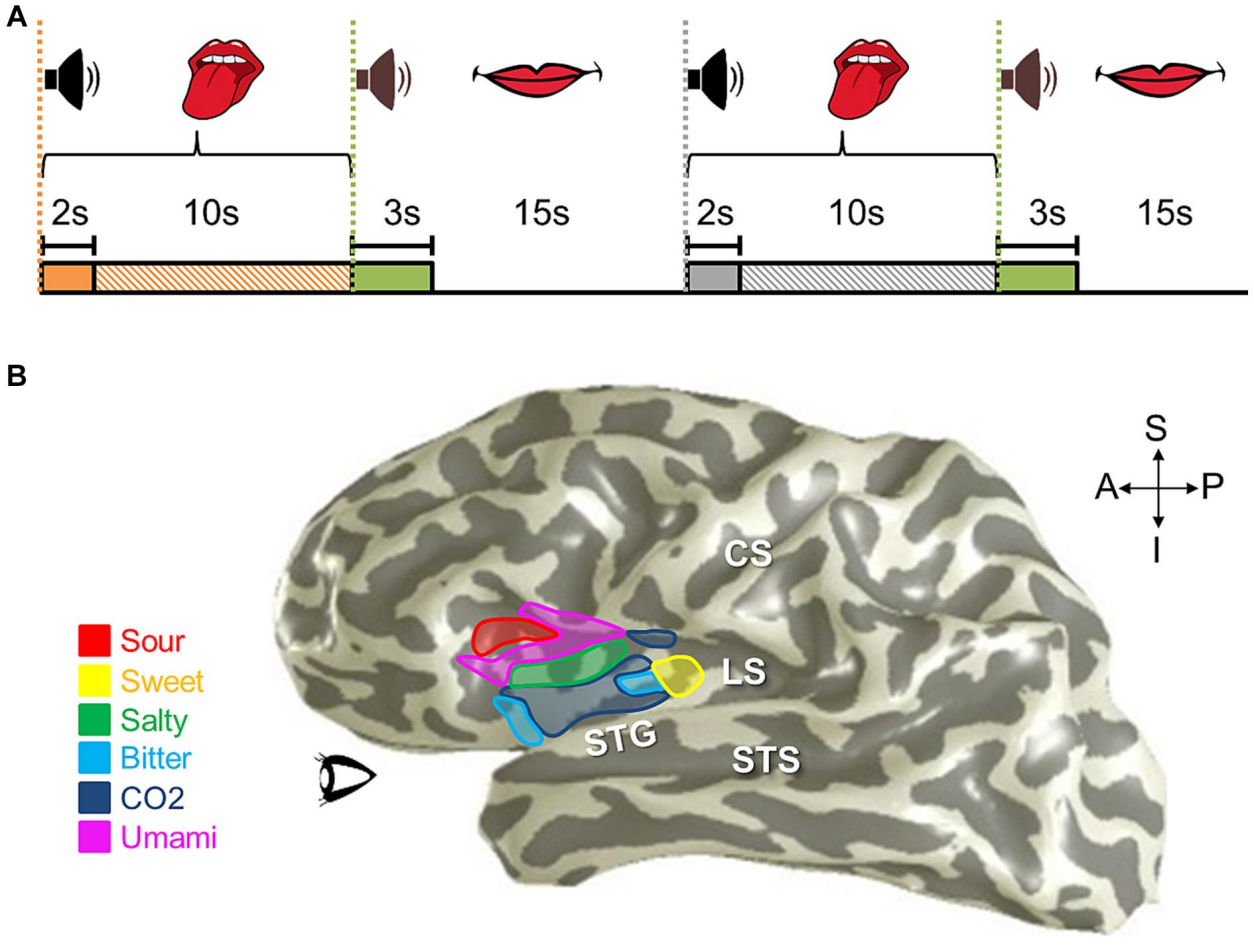
One dimension of gustatory field maps has been identified for taste representations in human insular cortex. **(A)** The schematic displays an example organization of one taste-and-rinse gustatory stimulus cycle, based on the gustatory mapping paradigm presented in Prinster et al. (2017). The stimulus begins with an auditory cue (black speaker) that a taste-test solution (orange) is about to be delivered. The tastant is delivered via an injection for 2 s (solid orange bar) and then held in the mouth for tasting for an additional 10 s (orange striped bar). Full tasting time (open mouth icon) includes injection time and time the solution is held in the mouth (12 s in this example). A second auditory cue (brown speaker) next signals the start of the swallowing period (green bar), which is followed by a longer rest period (closed mouth icon). After each test solution, water (gray) is used in the same paradigm for rinsing between taste tests. The cycle then repeats with the next tastant. FMRI data acquisition is continuous, with a TR = 2 s in this example. **(B)** Colored ROIs denote estimated locations for selective cortical responses based on group-averaged data from Prinster et al. (2017) for the six primary receptor-mediated tastants: sour (red), sweet (yellow), salty (green), bitter (cyan), CO_2_ (navy), and umami (magenta). ROIs are overlaid on a 3-D inflated rendering of a left hemisphere, with gyri marked in light gray and sulci in dark gray. Even with some overlap of tastant responses, a topographical organization of these six principal tastes can be seen. CS, central sulcus; LS, lateral sulcus; STG, superior temporal gyrus; STS, superior temporal sulcus. Anatomical-directions legend: S, superior; I, inferior; A, anterior; P, posterior.

Very recent updates from a handful of studies have measured topographical representations of basic taste stimuli at the putative location of a primary gustatory cortex in human along the insular cortex and adjacent operculum (Prinster et al., 2017; Chikazoe et al., 2019). These studies utilized high-field (7 T) fMRI to delineate the some or all of the five key tastes plus the perception of CO_2_ using both traveling-wave and multivoxel pattern analysis to examine the various taste responses (Figure 14A). Although the exact pattern of activation of the insula by specific taste categories appears to be somewhat variable across subjects, these studies do demonstrate a consistent topographical mapping of gustatory information in this region (Figure 14B). This is a very exciting finding that supports CFMs as a fundamental organizing principle in the chemical senses as well. It is important to note, however, that these gustatory measurements span a broad area of the cortical sheet, likely larger than the surface area of V1, with broad and scattered regions of specific basic tastes. Whether this entire region contains just one gustatory field map (GFM) or, more likely, multiple GFMs each subserving unique gustatory computations remains to be seen. In addition, these representations of specific taste types compose one dimension of purported GFMs. A second dimension could arise from a range of parameters, from low-level properties like molecular parameters (other than those contributing to the first basic-taste dimension) or taste intensity or higher-level properties such as palatability/hedonistic value.

#### 4.4.2 Olfactory topographies

The perception of odors similarly begins in mammals with the recognition of odorant molecules by a diverse set of approximately 1,000 different odorant receptor types (Vassar et al., 1994; Fleischer et al., 2009; Yeshurun and Sobel, 2010b). These seven-transmembrane receptors are expressed on the olfactory sensory neurons (OSN; also known as olfactory receptor neurons) in the olfactory sensory epithelium (OE) along the posterior/superior aspect of the nasal sinuses (Buck and Axel, 1991). Each OSN expresses a single olfactory receptor gene, and each gene has its own contiguous expression area within the OE that overlaps with those of its neighbors (Vassar et al., 1993; Miyamichi et al., 2005). Each olfactory receptor not only can interact with a diverse set of odorants, but also demonstrates high specificity for its specific olfactants. Thus, subtle alterations in the structure of an odorant molecule can often cause major changes in the perceived odor. In fact, humans are astonishingly adept at olfactory discrimination, with one study suggesting that they can distinguish more than one trillion olfactory stimuli (Ressler et al., 1994; Bushdid et al., 2014). This sensory prowess likely reflects the vast options for combined outputs from ∼400 different subtypes of olfactory receptors (Gilad and Lancet, 2003; Auffarth, 2013). Since the genome of olfactory receptor subtypes varies by about 30% across individuals, each person’s olfactory epithelium is composed of a potentially entirely different set of olfactory receptor genes that may even produce unique olfactory perception in each individual (Menashe et al., 2003; Keller et al., 2007; Secundo et al., 2015).

OSNs are bipolar cells that express their olfactory receptors on their apical dendrites in the nasal cavity and project their axons through the cribriform plate of the skull to synapse with the mitral and tufted cells of the olfactory bulbs. OSNs with the same olfactory receptor synapse onto a single specific glomeruli within an olfactory bulb (Figure 13B; Mori et al., 1999; Imai et al., 2010; Murthy, 2011; Francia and Lodovichi, 2021). This convergence of OSN projections with matching receptor types in the glomeruli is facilitated by the expression of the same olfactory receptors at both the OSN’s dendrites in the OE and their synapses in the olfactory bulbs (Mori et al., 1999; Cho et al., 2009; Lodovichi, 2020). A discrete sensory map is thus formed here around the identity of the odorant receptors (Mombaerts et al., 1996; Luo and Flanagan, 2007). Ma et al. suggest that the organization of the glomeruli in mice is then based on loosely grouping together glomeruli tuned to specific molecular properties—such as esters, ketones, etc.—with similar tuning properties (Ma et al., 2012). Such a “tunotopic” map may aid odor discrimination by enhancing the contrast among similar odors. Ultimately, each odorant produces a unique pattern of activity across the glomeruli in the olfactory bulbs that is stable over at least several months (Kato et al., 2012). While the majority of studies of these topics rely on various animal models, these stages of olfactory processing appear to be highly conserved across mammals including humans (Zelano and Sobel, 2005; Echevarria-Cooper et al., 2022).

Despite our understanding of many aspects of this early olfactory processing, there has been a lot of difficulty with determining what organization may be present within the next steps of olfactory processing in the mammalian piriform cortex. For many years, piriform cortex was generally thought to lack topographical organization (Murthy, 2011; Sosulski et al., 2011). Without clear evidence for such topography, several studies have proposed that these neural computations rely on experience-based plasticity across the lifespan to develop and update the necessary olfactory processing circuits (e.g., Babadi and Sompolinsky, 2014; Schaffer et al., 2018; Hiratani and Latham, 2020; Schoonover et al., 2021). However, a very recent study in mice has utilized cutting-edge neuroanatomical techniques to map the brain-wide projections among thousands of individual neurons in the olfactory bulb and piriform cortex, a much larger sample than prior work was able to achieve (Chen et al., 2022). Their results suggest that the olfactory cortex connectivity is in fact spatially structured. An olfactory bulb neuron (i.e., mitral cell) projects both to a particular location along the anterior–posterior axis of piriform cortex and to matched and functionally distinct cortical targets outside of the piriform. In addition, single neurons from the piriform project to the same extra-piriform targets that their matched olfactory bulbs neurons project to. This triadic circuit organization that routes olfactory information to functionally distinct regions of cortex is quite compelling, as it positions olfaction as having a similar framework for coordinated, parallel processing pathways of sensory information as we see in the other sensory systems (Chon et al., 2020; Imamura et al., 2020; Francia and Lodovichi, 2021; Chen et al., 2022). It remains to be seen whether such olfactory representations also compose CFMs, cloverleaf clusters or dorsal/ventral processing streams and whether these measurements in mice are applicable to humans as we expect. Based upon the current findings in gustatory cortex, one avenue to investigate potential olfactory field maps (OFMs) would be to search for topographical representations of olfactant molecular properties, concentration/intensity, or palatability.

## 5 Discussion

Common schemes of topographical organization are thus emerging across human sensory systems. Visual and auditory cortices are compartmentalized into CFMs that are themselves arranged on a larger scale into cloverleaf clusters. This fundamental organization likely provides a structure for the complex processing and analysis of inputs from their peripheral sensory receptors. Somatosensory cortex in human shares similar parallel processing pathways as those two senses as well as a loose division into dorsal and ventral streams. Ongoing investigations into the details of the somatotopic maps in human S1 and S2 are beginning to reveal the details of the SFMs in humans. Despite the differences in the discrete properties of the molecular stimuli in the chemical senses, recent studies in human and animal models demonstrate that gustation and olfaction may utilize similar topography as well. Knowledge of how these topographical representations are organized across cortex provides us with insight into how our conscious perceptions are created from our basic sensory inputs. The detailed examination of these CFMs and clusters in individual humans and across species can be applied to the careful analysis of the computational stages of sensory. In addition, studying how these representations change during development, trauma, and disease can serves as an important tool for developing improvements in clinical therapies and rehabilitation for sensory deficits.

## Author contributions

AB and BB conceived of the concept and developed the discussion. AB wrote the manuscript. BB revised it. All authors contributed to the article and approved the submitted version.
